# Heat shock protein 60 and cardiovascular diseases: An intricate love‐hate story

**DOI:** 10.1002/med.21723

**Published:** 2020-08-17

**Authors:** Indumathi Krishnan‐Sivadoss, Iván A. Mijares‐Rojas, Ramiro A. Villarreal‐Leal, Guillermo Torre‐Amione, Anne A. Knowlton, C. Enrique Guerrero‐Beltrán

**Affiliations:** ^1^ Tecnologico de Monterrey, Escuela de Medicina y Ciencias de la Salud, Medicina Cardiovascular y Metabolómica Monterrey Nuevo León México; ^2^ Methodist DeBakey Heart and Vascular Center, The Methodist Hospital Houston Texas; ^3^ Veterans Affairs Medical Center Sacramento California USA; ^4^ Department of Internal Medicine, Molecular and Cellular Cardiology, Cardiovascular Division University of California Davis California USA; ^5^ Department of Pharmacology University of California Davis California USA; ^6^ Tecnologico de Monterrey, Hospital Zambrano Hellion, TecSalud, Centro de Investigación Biomédica San Pedro Garza García Nuevo León México

**Keywords:** heart diseases, heart failure, heat shock proteins, immunity, therapeutic

## Abstract

Cardiovascular diseases (CVDs) are the result of complex pathophysiological processes in the tissues comprising the heart and blood vessels. Inflammation is the main culprit for the development of cardiovascular dysfunction, and it may be traced to cellular stress events including apoptosis, oxidative and shear stress, and cellular and humoral immune responses, all of which impair the system's structure and function. An intracellular chaperone, heat shock protein 60 (HSP60) is an intriguing example of a protein that may both be an ally and a foe for cardiovascular homeostasis; on one hand providing protection against cellular injury, and on the other triggering damaging responses through innate and adaptive immunity. In this review we will discuss the functions of HSP60 and its effects on cells and the immune system regulation, only to later address its implications in the development and progression of CVD. Lastly, we summarize the outcome of various studies targeting HSP60 as a potential therapeutic strategy for cardiovascular and other diseases.

**Abbreviations**
ADP: adenosine diphosphate
AMI: acute myocardial infarction
APC: antigen presenting cell
ApoB‐100: apolipoprotein B‐100
ATP: adenosine triphosphate
Bak: Bcl‐2 homologous antagonist/killer
Bax: Bcl‐2 homologous X protein
BCR: B cell receptor
BNP: brain natriuretic peptide
CAD: coronary artery calcification score, coronary artery disease
CAF: chronic atrial fibrillation
CCR2: C‐C chemokine receptor
COX‐2: cyclooxygenase‐2
DAMP: damage associated molecular pattern
DC: dendritic cell
DCM: dilated cardiomyopathy
eGFP: enhanced green fluorescent protein
ERK: extracellular signal regulated kinase
HF: heart failure
HFpEF: heart failure with preserved ejection fraction
HMGB1: high mobility group box 1
HSA: human serum albumin
HSF‐1: heat shock transcription factor 1
HSP: heat shock protein
HSP10: heat shock protein 10
HSP27: heat shock protein 27
HSP60: heat shock protein 60
HSP70: heat shock protein 70
ICAM‐1: intercellular adhesion molecule 1
IFN‐γ: interferon γ
IGF‐1: insulin like growth factor‐1
IL: interleukin
iNOS: inducible nitric oxide synthase
IRAK‐1: IL‐1R associated kinase
JNK: Jun N‐terminal kinases
LDL: low density lipoprotein
LDLR: low density lipoprotein receptor
LPS: lipopolysaccharide
MAPK: MAP kinase
MDA: malondialdehyde
MHC‐I: major histocompatibility complex‐I
MHC‐II: major histocompatibility complex‐II
miR‐1: microRNA‐1
mRNA: mitochondrial RNA
mtHSP60: mitochondrial HSP60
MyD88: TLR‐4 myeloid differentiation protein 88
NF‐κB: nuclear factor κB
NK: natural killer
NO: nitric oxide
NOS‐2: nitric oxide synthase 2
NSTEMI: non ST‐elevation myocardial infarction
oxLDL: oxidized LDL
PAH: pulmonary arterial hypertension
PAMP: pathogen associated molecular pattern
PCNA: proliferating cell nuclear antigen
PRR: pattern‐recognition receptor
ROS: reactive oxygen species
RT‐PCR: reverse transcriptase polymerase chain reaction
siRNA: small interfering RNA
STEMI: ST‐elevation myocardial infarction
TCR: T cell receptor
TGF‐β: transforming growth factor β
Th1: type 1 T helper cell
Th17: type 17 T helper cell
Th2: type 2 T helper cell
TIR: Toll/IL‐1 receptor
TLR: toll‐like receptor
TNF‐α: tumor necrosis factor α
TRAF6: TNF receptor associated factor 6
Treg: regulatory T cells
TRIF: TIR‐domain‐containing adapter inducing interferon b
T cell: T lymphocyte
VCAM‐1: vascular cell adhesion molecule‐1
VSMC: vascular smooth muscle cell

## 1 INTRODUCTION

The cardiovascular system, comprising the heart and blood vessels, has a central role in human physiology, with its main contribution being the continuous supply of nutrients and blood to the remaining tissues to support their metabolic activities. Often regarded as a pump, the heart is a highly specialized muscle that contracts in a sustained and rhythmic fashion to keep blood flowing throughout an entire circuitry of arteries and veins that branch out to reach peripheral tissues and allow for nutrient and oxygen diffusion. A constant heartbeat is indispensable to maintain the body alive, and thus the pump is ever active. This nonstop agenda demands that the heart is also provided with an effective nutrient supply of its own. The net result of the process hitherto described is metabolically favorable because of the pump's high efficiency, requiring ~4% of the cardiac output at rest to comply with its duties.^1^

The heart is an organ that runs preferentially on oxidative phosphorylation to satisfy its energy demands, so that it is highly dependable on continuous blood flow and even a minor shortage due to obstruction may greatly impair its contractility and lead to death. To avert the potential threat of substrate deprivation, cardiac tissues rely on protective mechanisms including a collateral circulation, an antioxidant system and other intracellular stress responses that safeguard their integrity. Among these defense schemes, the heat shock protein (HSP) response is a remarkably preserved evolutionary feature that is present both in prokaryotic and eukaryotic organisms. The great heterogeneity of responses elicited by this system is also the result of the sizable number of members this protein family has. HSPs were originally identified due to their prompt activation after abrupt increases in temperature and they were theorized to assist cells in adapting and surviving under these circumstances.^2^

Heat shock protein 60 (HSP60), a prominent protein of the HSP family, has been exhaustively studied because of its cardioprotective properties, which include processing misfolded proteins, antiapoptotic activity, and dynamization of key transcription factors for mitochondrial biogenesis and calcium handling. Paradoxically, this very same protein may also be a source of inflammation not only for the tissues it stems from, but also for peripheral ones. It has been demonstrated that it stimulates both innate and adaptive immunity behaving as a damage associated molecular pattern (DAMP) eliciting robust immune responses triggering some mechanisms of inflammation, an integral part of cardiovascular disease (CVD) pathophysiology.^3^ Evidence has unearthed interesting patterns in HSP60 levels in various CVDs and the consequences are quite relevant for disease progression. To understand this powerful notion, we will begin by providing a panoramic view of HSP60 in cell physiology followed by insight to the dual role it holds to regulate immune responses by either eliciting or mitigating inflammation. Finally, we will address the circumstances under which this protein may turn against self to wreak havoc and promote tissue damage as a process directly related to CVD. We will conclude this review by highlighting the implications these findings have for the development of different potential therapeutic modalities in cardiovascular and many other diseases applicable.

## 2 HEAT SHOCK PROTEIN 60

HSPs are a large family of intracellular proteins that received their name after the discovery that their upregulation responds to heat shock, and their overall purpose is to grant protection against this otherwise menacing condition (Table 1). They are also called molecular chaperones, a name often used interchangeably with HSP, however some HSP have no function in chaperoning and act as moonlighting proteins with secondary functions which will be further discussed. Traditional nomenclature designated each of its members with the family's acronym followed by their respective molecular weight. A decade ago, Kampinga et al.^4^ designated a new classification to HSPs where HSP60 received the name of HSPD1, however this classification is not popularly used. Interestingly, the chaperones of 60 kDa are classified into two different groups chaperonins, a name used interchangeably with HSP60. Group I of chaperonins can be found in the mitochondria, its primary location, and chloroplasts of eukaryotes but can have other different locations such as the cytosol, cell membrane, and cell surface.^5^ They can also be found in prokaryotic cytoplasm.^6^ Group II of chaperonins are mainly located in cytoplasm of eukaryotes as well as in archaebacterial microorganisms.^6^ However, in this review we will be focusing specifically on Group I.

**Table 1:** Different mammalian HSPs and their overall physiological function

Heat shock protein	Overall functions
HSP27	As part of the small heat shock protein family (sHSP), this protein participates as a chaperone, in cytoprotection, antiapoptosis and antioxidation^202^, ^203^
HSP60	Along with HSP10, this protein has chaperone activity, immunoregulation and cytoprotection^204^
HSP70	This protein acts as a chaperonin and participates in autophagy, antiapoptosis and cytoprotection^205^
HSP90	This protein aids in chaperonin functions, intracellular signaling, pro‐apoptosis and cell‐cycle control^206^, ^207^, ^208^
HSP110	It functions as a chaperone and provides a thermotolerant effect for cells^209^

Apart from being a very well phylogenetically preserved protein, HSP60 is present in a wide array of organisms, including fungi, plants, bacteria, and mammals.^7^, ^8^ Due to this fact, an important degree of homology between species exists. For prokaryotic organisms, almost 75% of sequence identity is shared, with some epitopes reaching 90%; a 50% match is observed between bacterial and mammalian HSP60.^8^, ^9^, ^10^

These structural similarities became greatly helpful when studying the function of HSP60 in cell biology. Most of the knowledge we have about human HSP60 came from previous observations of several prototypes that have since been used; these include bacterial homologues such as *Escherichia coli* GroEL, *Chlamydia trachomatis* HSP60 GroEL‐like, *Mycobacterium tuberculosis* HSP65 and HSP60 of fungi such as *Aspergillus* spp., *Candida* spp., and *Histoplasma* spp.^10^, ^11^, ^12^, ^13^ From studying these units, we now know that HSP60 is mainly located inside the mitochondria for protein‐folding purposes, preventing the aggregation of misfolded polypeptide clients while assisting during their refolding.^14^, ^15^ In mammalian cells, around 75%–80% is located within this organelle while 15%–20% has an extramitochondrial location.^16^ The main extramitochondrial hubs for HSP60 are the cytosol, endoplasmic reticulum, and nucleus, wherein it lends its chaperonin services.^16^ However, cell surface location of HSP60 is found especially under pathological conditions and extracellular localization of HSP60 is associated with proinflammatory changes and apoptosis and marks the cell for detection by the immune system.^17^

Recently, it has been suggested that in the extracellular space HSP60 can be released via the exosomal pathway and during necrosis via passive leakage, implying that both processes may play a role in death signaling.^18^ A study has reported results indicating that under stress conditions, there is a release of ubiquitinated HSP60 through exosomes by adult cardiac myocytes.^19^ However, not always do exosomes leak HSP60. Another study demonstrated that exosomal HSP60 seems to be stable within the exosomes released under various conditions.^20^ Due to the unique characteristics of exosomal HSP60, it seems to be a promising tool as a prognostic marker for many other diseases: levels of exosomal HSP60 vary according to the pathological condition, it acts as a unique fingerprint of the cell that releases, reflects the functional status of the cell, and circulates all around thereby can be sampled easily,^20^, ^21^ nevertheless, the fate of these vesicles remains to be fully understood. To further understand the different roles of HSP60 in cell physiology and as a potent immune system activator or trigger, the assembly of the functional unit and structure will be described in detail in the following section.

## 3 STRUCTURAL CHARACTERISTICS

HSP60 is encoded within the nuclear genome and is expressed in the cytosol as a precursor termed naive HSP60, which differs from mitochondrial HSP60 (mtHSP60). The former carries a 26 amino acid (aa) sequence which serves as a mitochondrial import segment necessary for its transport into the organelle.^22^ Once inside, it is cleaved rendering the fully mature mtHSP60.^22^ Mitochondrial import segment also aids in naive HSP60 stabilization and makes it more resistant to denaturant conditions, properties that are lost in mtHSP60, however said resulting instability seems to be important for its physiologic role in the mitochondria.^22^ Nonetheless, as naive HSP60 in aqueous solution, a study has demonstrated that its structure can be found in stable heptamers and tetradecamers at different concentrations.^23^

In its minimal functional unit, HSP60 exists as a single ring structure forming a heptameric toroid, although it is mostly present as two‐stacked heptameric rings which form a central enclosure where proteins are folded in an ATPase‐dependent activity.^24^ Within the hollow, central cavity from these structures, the hydrophobic and flexible C‐terminal of each subunit protrude into one another.^25^ These domains play a major purpose in protein folding, as evidenced by the resulting impairment in cell growth stemming from mutations related to them.

In speaking about HSP60's quintessential role as a chaperonin, it should be stated that it is known to occur as a cooperative effort that requires another HSP. HSP10 co‐chaperonin, a related mitochondrial chaperone, is necessary for said purpose, whereby it functions as a cover for the developing toroidal canal. In a similar fashion, HSP10 also assembles into heptameric structures.^26^ Unlike their bacterial homologues GroEL and GroES, the mammalian chaperones HSP60 and HSP10, respectively, form a single ring structure, an adenosine triphosphate (ATP)‐dependent chaperone system inside the mitochondria, where the role of HSP60 is essential in protein folding and matrix protein refolding, being upregulated during mitochondrial stress.^27^ This is possible due to the fact that when in an adenosine diphosphate‐bound state, the HSP60–HSP10 complex has very weak interactions that allow for a single ring structure to assemble, while the GroEL–GroES complex shows strong interactions that require a second trans ring for its correct functioning.^24^ Regarding the different conformational states in which HSP60 complexes may be present, Ishida et al.^28^ proposed a reaction cycle for the HSP60–HSP10 complex where on one hand, in the absence of nucleotides, HSP60 has a single ring structure and on the other, a double ring structure when associated with HSP10 in the presence of ATP. Okamoto et al.^29^ also proposed a GTPase activity in HSP60 mediating protein folding along with HSP10. The details of HSP60 structure under different cell conditions remains to be completely resolved.

## 4 PHYSIOLOGICAL ROLES

A relevant characteristic of mammalian HSP60 that sets it apart from its chloroplast and bacterial homologues is its cochaperonin specificity. According to Levy‐Rimler et al., not only does mammalian HSP60 associate into tetradecamers or monomers depending on whether protein concentrations are high or low, respectively, but it also functions exclusively with its own specific mitochondrial 10 kDa cofactor HSP10, while other HSP60 homologues can work with any cognate of HSPE1 (HSP10).^30^ The chaperonin also weaves tight‐knit networks with other HSPs beyond HSP10. An example of these relations is best represented with the HSP70 system, which are chaperones that protect polypeptides from misfolding and aggregation during production and release from the ribosomal exit site, after which HSP60 chaperonins take care of any collapsed folding intermediates.^31^ An interesting example that illustrates how HSP60 contributes in proteostasis has been demonstrated in vitro in a study conducted by Mangione et al. They observed that co‐incubation of amyloid β (Aβ) and the chaperonin inhibited Aβ aggregation possibly by blocking pathways of fibrillogenesis, the basis of the pathophysiology of Alzheimer's disease. Therefore, the protective mechanisms of HSP60 extend outside the simple protein folding realm and aid in proteostasis in pathological settings as well.^32^

As HSP60 interacts with HSP70 to form an HSP60–HSP70 complex, it allows the transportation of proteins across the cell. A major client for this chaperoning complex is mitochondrial transcription factor A, a protein with a pivotal role in the homeostasis of the organelle from which its name derives, primarily by fine‐tuning the expression of genes associated with mitochondrial biogenesis, Ca^2+^ handling and regulation of reactive oxygen species (ROS) production.^33^ Mitochondrial transcription factor A starts its exodus from the nucleus to the mitochondrial matrix after binding to the HSP70 moiety of the chaperoning complex, and upon reaching its destination, release is dependent on Lon protease binding to HSP60.^33^ It has also been demonstrated in mice that significant periods of exercise and training can increase HSP60 levels in the bloodstream with a concomitant increase in peroxisome proliferator‐activated receptor ɣ coactivator 1α (PGC1α) expression indicating greater regulation of mitochondrial biogenesis with an increase in mitochondria, a key step in adaptation of skeletal muscle in endurance training.^34^ Thus, under physiological conditions HSP60 can follow different patterns of distribution inside and outside the cell regulating essential adaptive mechanisms in muscle fibers.

In apoptosis, HSP60 interacts with a number of proteins related to proapoptotic as well as antiapoptotic events such as procaspase‐3,^35^, ^36^, ^37^ survivin,^38^ cyclophilin D,^39^ p53,^38^ and Bcl‐XL, Bcl‐2 homologous antagonist/killer (Bak) and Bcl‐2‐associated X protein (Bax).^40^ When referring to HSP60 as an ally to cell systems, as an antiapoptotic molecule, it sequesters both Bax and Bak; HSP60 decreases in front of high levels of Bax associated with mitochondrial membrane.^41^ Heat shock stress is known to hinder both inner and outer mitochondrial membrane potential, ultimately leading to dysfunction of the organelle and triggering cell death. HSP60 downregulation using small interfering RNA (siRNA) has been associated with higher apoptotic, but not necrotic activity. Moreover, this finding was related to an increase in mitochondrial Bax and disruption of mitochondrial outer membrane permeabilization but not inner membrane permeabilization, which further highlights HSP60's role as a sentinel for Bax, which when absent, may account for the proapoptotic protein's pore‐inducing activity in the mitochondria and the observed increase in programmed cell death.^42^ However, during cellular stress, mtHSP60 may induce apoptosis by interacting directly with procaspase‐3 and promoting its activation to caspase‐3.^36^ It is also suggested that the cytosol accumulation of HSP60 may or may not result from mitochondrial release, as it depends on the nature of the stressing stimulus.^25^ Overall, HSP60 favors more cell survival mechanisms than apoptosis, therefore it has been suggested that high levels of this protein found in different types of cancer correlates with tumor cell growth.^43^

## 5 HSP60 IN NONIMMUNE SOMATIC CELLS

The following segment describes some of the roles that have been suggested HSP60 plays within nonimmune somatic cells. The following are activities different to the classically described for the chaperonin, and thus further evidence from other research groups will be needed to solidify these findings and state them as canon functions of the protein.

Apart from its chaperoning and protein folding roles and immune response stimulating capacity, that will be described, HSP60 acts as a mediator for many other functions. For example, in vascular smooth muscle cells (VSMCs), it has been demonstrated that human and bacterial HSP60 induce proliferation.^44^ *Chlamydia pneumoniae* has mitogenic effects on VSMCs through regulation of HSP60 levels by increasing endogenous intracellular HSP60 expression levels.^44^ Higher levels of HSP60 unleashed proliferating cell nuclear antigen expression, a cell cycle protein, thus stimulating cell cycle progression and VSMCs proliferation.^44^ Also, HSP60 seems to be an important component during nuclear import, a key step for cell growth and replication, since it enables the internalization of transcription factors to the nucleus for further activation of genes related to the cell cycle. Modulation of nuclear protein import has been proposed as a possible mechanism through which human HSP60 mediates cell proliferation by regulating expression levels of importin‐α, importin‐β, and Ran while mtHSP60 induces nucleoporin (Nup62, Nup153) expression levels. As components of the nuclear pore complex, they catapult the cell into an enhanced metabolic state.^44^, ^45^ These implications cast light on the pathophysiology of diseases such as hypertension and atherosclerosis, where VSMCs proliferation is an integral component.

In endothelial cells, ATP synthase is yet another of the many polypeptide clients of HSP60 and Alard et al.^46^ were able to observe that the chaperonin aids in intracellular pH regulation by means of promoting proper ATP synthase activity. This is linked to a certain extent to previous reports by Jamin et al.^47^ stating that HSP60 is a target for antiendothelial cell antibodies, inducing apoptosis in vasculitis. It also seems that HSP60 induces nitric oxide synthase‐2 (NOS‐2) and cyclooxygenase‐2 (COX‐2) expression in macrophages and endothelial cells. Both enzymes participate in inflammation processes, COX‐2 needed for the conversion of arachidonic acid to prostaglandin H_2_ and NOS‐2 for nitric oxide (NO) production.^48^ While COX‐2 expression in macrophages was noticeable at 4 h, in endothelial cells the expression was delayed for 16 h.^48^ The expression of NOS‐2 induced by HSP60 also presented similar time frames in both cell types.^48^ In another in vitro study, HSP60 of *Porphyromonas gingivalis* was able to downregulate expression levels of VE‐cadherin and eNOS in human endothelial cells after coincubation.^49^ Endothelium‐derived NO participates in vascular tone regulation and survival and migration of endothelial cells. VE‐cadherin, an adhesive molecule that regulates cell permeability, migration, and assembly in angiogenesis, is also important for cell integrity. The mechanisms through which HSP60 is able to regulate the levels of these proteins is not clear enough yet.^49^

In cardiomyocyte cells, it is well established that toll‐like receptors (TLRs) are expressed on the cell membrane, specifically TLR4 and TLR2.^50^, ^51^ Under pathologic conditions, expression levels of these receptors increase as they recognize extracellular HSP60 released by stress stimuli.^3^ This interaction triggers inflammatory cytokine production such as tumor necrosis factor ɑ (TNF‐ɑ) and interleukin 6 (IL‐6) by means of the activation of the nuclear factor‐κB (NF‐κB) signaling pathway and TLR4‐myeloid differentiation protein 88 (MyD88)‐p38 and upregulates expression levels of TLR2 and TLR4 through the TLR4‐MyD88‐c‐Jun N‐terminal kinases (JNK)/NF‐κB pathway.^52^

Among the possible neurological pathways mediating the expression of HSPs, it has been determined that cholinergic activation via muscarinic acetylcholine receptors in hippocampal neurons leads to an increase in heat shock transcription factor 1 (HSF1)‐Ser326 modification which leads to the activation of this transcription factor, with a dose and time‐dependent increase in HSPs including HSP70, HSP90, and HSP60.^53^

In various types of cancer, HSP60 appears to have a role in diagnosis, prognosis and prevention.^54^, ^55^, ^56^, ^57^ Different cancers present increased levels of HSP60 and seems to be linked to prosurvival mechanisms, such as uncontrolled proliferation and loss of replicative senescence. The apoptotic threshold is increased conferring a cytoprotective function to HSP60.^43^ As in pre‐neoplastic stages and invasive cancer overexpression and intracellular accumulation of HSP60 can be found, chlamydial HSP60 found in abundance in chronic infection could trigger pathogenicity for cervical cancer by cross‐reactivity with immune cells, an interesting hypothesis stated by Di Felice and colleagues.^58^ This could be achieved by inflammation, reactivity against self HSP60 located on the surface and disruption of apoptosis and senescence.^58^, ^59^ Nonetheless further experimentation is needed to confirm this notion.

In addition to the already numerous roles so far described in which HSP60 is involved, recent reports suggest that its apoptotic‐related activities may be important for embryonic development. By using Cre‐LoxP HSP60 transgenic models, it has been observed that mouse embryos expressing HSP60 were born with congenital atrial septal defects, severe hemorrhage and evidence of increased myocyte apoptosis, which may account for the inborn structural damages aforementioned.^60^ As a result of the cardiovascular burden, heart failure (HF) ensued at postnatal day 1 and was the ultimate cause of death in these transgenic mice.^60^ During the embryonic stage, apoptosis plays a fundamental part for development and is a regulated process^60^ Even though HSP60 transgenic mice demonstrated increased apoptosis and congenital atrial septal defects, it is unclear if a relationship exists between HSP60 induction and atrial septal defects.^60^ The study speculated that atrial septal defects seen in HSP60 transgenic mice is a consequence of incomplete development and increased apoptosis.^60^ However, further research is needed in this regard due to its importance and its probable relationship on newborn heart defects.

The proteomic profiles of the cardiac chambers have proven to be heterogenous from one another, an interesting feature about HSP60. It has been reported that basoapical proteomic differences exist within the left and right ventricles.^61^ In a study, an increase in five proteins, one of them being HSP60, was observed in the apical region of the left ventricle, which could have great implications for pathophysiologic processes occurring preferentially in said area, such as ischemic injury during acute myocardial infarction (AMI).^61^ However, right ventricular baso‐apical proteomic profile did not exhibit this increase in HSP60.^61^

Thus, HSP60 has various relevant roles apart from the well analyzed and studied task as a chaperone and interacts with a number of proteins, which are key for proper cellular functioning. However, as the synthesis of HSP60 is upregulated and liberated by stress stimuli in different types of cells, it is also one of the main culprits in the pathophysiology of some CVDs given the downstream effects of HSP60. In the cardiovascular system, the release of HSP60 into the bloodstream is a process undertaken directly by cardiomyocytes via exosomes rather than the classic Golgi apparatus pathway under stress conditions where the chaperone protein remains tightly attached to the exosome membrane to eventually be released into the extracellular space.^19^ Ergo, it is important to comprehend the intricate mechanisms through which HSP60, an immunogenic protein, interacts with the innate and adaptive immune systems as an elicitor of inflammation. This phenomenon will be discussed below for its relevance in the progression of CVDs.

## 6 HSP60 IN INNATE IMMUNITY

**Figure 1:**
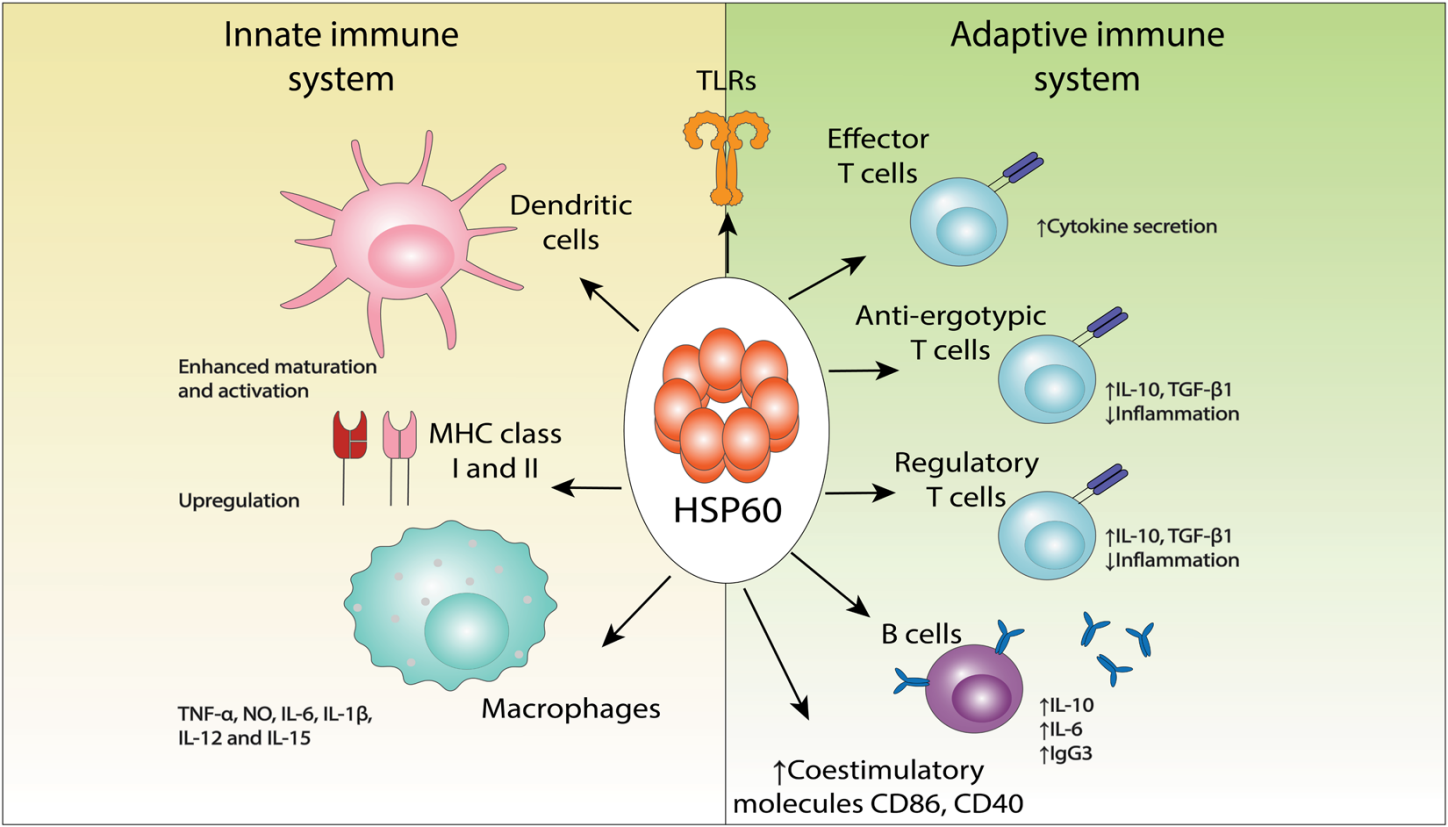
Summary of key immune functions of HSP60. HSP60 is recognized by TLRs in both macrophages and DCs eliciting inflammatory responses. This is achieved through different mechanisms including antigen‐presentation by APCs, increased maturation of DCs, cytokine secretion by macrophages, and upregulation of the costimulatory molecules of MHC‐I and MHC‐II, CD86 and CD40. HSP60 is also recognized by TCRs and can favor inflammation through effector T cells or suppress inflammation through anti‐ergotypic and regulatory T cells. HSP60 peptides recognized by B cells can elicit anti‐inflammation via IL‐10 secretion or proinflammation via the release of HSP60 antibodies. As such, immune effects elicited by this protein are variable and subjected to local concentrations. APC, antigen presenting cell, IL, interleukin; MHC, major histocompatibility complex [Color figure can be viewed at wileyonlinelibrary.com]

Previous studies have demonstrated that HSP60 has proinflammatory properties when interacting with innate immunity cells, for example, macrophages and dendritic cells (DCs). DCs possess membranous extensions that inspect the area covered and are very phagocytic. They participate both in the innate and adaptive immunity when activated which allow them to rapidly intake the antigen and secrete cytokines (Figure 1). The molecules or antigens recognized by these cells are called pathogen‐associated molecular patterns (PAMPs), DAMPs, and alarmins; PAMPs being more prominent in microbes, whose structural components are characteristic as they are not found in the host.^62^ Examples of PAMPs include flagellin and lipopolysaccharides (LPS), while alarmin examples include high mobility group box 1 (HMGB1) protein and HSPs, in which the latter also displayed DAMPs activities when released as an endogenous response.^3^ When recognized and bound to pattern‐recognition receptors (PRRs) on innate defense cells, the ligand‐receptor complex is internalized, and cell activation occurs mounting an inflammatory response immediately.^63^ This is in contrast to the adaptive immunity, where instead of performing their effector functions, they undergo proliferation and expansion. These PRRs however, are expressed in a wide range of cells, including effector cells (DCs, macrophages, lymphocytes, and neutrophils) and nonimmune somatic cells (cardiomyocytes, endothelial cells, epithelial cells, etc.).^62^ Bacterial and human HSP60, acting as DAMPs, elicits and triggers a rapid release of NO, TNF‐α, IL‐1β, IL‐6, IL‐12, and IL‐15 from macrophages.^64^ It also has the ability to upregulate costimulatory molecules of major histocompatibility complex class I (MHC‐I) and II (MHC‐II), CD86 and CD40, and promote the maturation of DCs and the capacity of antigen‐presentation in antigen presenting cells (APCs).^64^

When found in the extracellular space, HSP60 may play both proinflammatory and anti‐inflammatory roles depending on its interactions with cell‐surface receptors including TLRs, while it may also bind to other proteins during an immune response to assist in their presentation to lymphocytes.^65^ TLRs are PRRs present in various types of cells such as polymorphonuclear cells, mast cells, macrophages, DCs, T lymphocytes (T cells), natural killer cells, and B lymphocytes (B cells). They can have distinct localizations: TLR11, TLR6, TLR5, TLR4, TLR2, and TLR1 are found on the surface of the cell while TLR9, TLR8, TLR7, and TLR3 reside intracellularly.^66^ These receptors recognize different structures depending on the subtype of TLR. For example, TLR3 recognizes double‐stranded RNA while TLR7 binds to viral single‐stranded RNA. TLR2 binds to bacterial lipoteichoic acids and TLR4 recognizes bacterial LPS.^66^ TLRs participate in pathogen recognition and host defense and trigger innate immunity. They possess an extracellular domain and a cytoplasmic Toll/IL‐1 receptor (TIR) domain.^67^ After an adapter protein, for example, MyD88, binds to TIR, IL‐1R‐associated kinase (IRAK‐1) is engaged in the complex which is autophosphorylated and released from the complex. It posteriorly binds TNF receptor associated factor 6, which in turn phosphorylates NF‐κB and mitogen‐activated protein kinase (MAPK) to regulate the expression of chemokines and cytokines.^67^ TLR4 has predominantly been the focus in cardiomyocyte injury studies, and to a lesser degree, TLR2.^3^ HSP60 engagement to TLR4 and TLR2 as an alarmin protein is an important signaling event for cytokine production, including TNF‐α, IL‐8, and IL‐6,^68^ however it can prove to be deleterious in cardiac myocytes inducing cell injury and subsequent death. It has been demonstrated that HSP60, an already established ligand to TLR4, can induce apoptosis via NF‐κB pathway leading to TNF‐α production followed by apoptosis‐inducing factor release and cytochrome *c* from mitochondria, caspase‐3 activation, and DNA cleavage. To further investigate the role of HSP60 in apoptosis, participation of IL‐1β and TNF‐α, inflammatory cytokines produced by HSP60 and TLR4 engagement were analyzed and the results showed that DNA fragmentation occurred by TNF‐α and not by IL‐1β.^69^ Activation of TLR4 and TLR2, also via NF‐κB pathway, can generate impaired ventricular myocyte contractility as well.^70^ A study conducted by Wang et al.,^71^ indicated that the rat HSP60 gene has two NF‐κB binding sites. Human HSP60 gene analysis predicted three NF‐κB binding sites in the same study. After treating cells with TNF‐α, an upregulation in HSP60 expression was observed. As TNF‐α is an activator of the alternate pathway for NF‐κB activation, TNF‐α might be the culprit for increasing HSP60 in failing hearts.^71^

Microglia, members of the mononuclear phagocytic system, are the only cells in the central nervous system that express TLR4 on their surface and are thus responsive to the release of HSP60 in the extracellular matrix of neuronal tissues after cellular injury. A study performed by Lehnardt et al.,^72^ determined that HSP60 triggers apoptosis in microglia in a TLR4‐dependent fashion, as reported for other TLR4 expressing cells. Moreover, NO, which for neurons is cytotoxic, is also produced as a result of TLR4 activation mediated by HSP60, and accounts for yet another injurious mechanism. According to Swaroop et al.,^68^ HSP60 induces inflammation in N9 microglial cells, activating the downstream signaling of MAPK proteins (extracellular signal regulated kinase 1/2 [ERK1/2], JNK, and p38), and therefore the secretion of iNOX, COX‐2, and proinflammatory cytokines. To know which MAPK protein is specific to HSP60, Swaroop et al. reduced the levels of HSP60 with endoribonuclease‐prepared siRNA and all three MAPK proteins reduced their levels of activity meanwhile, microglia were treated with IL‐1β and rescued the effect of HSP60 endoribonuclease‐prepared siRNA in only ERK and JNK, assuming p38 MAPK is specific to HSP60.^68^ The same group performed another experiment to verify the specificity of p38 to HSP60.^68^ They used inhibitors U0126 (ERK), SP600125 (JNK), and SB203580 (p38) to block the three MAPK proteins as well as HSP60 cDNA clone.^68^ The expression of COX‐2 and inducible nitric oxide synthase (iNOS), both proinflammatory enzymes, was posteriorly assessed, as well as expression of IL‐6, TNF‐α, and MCP‐1, all of which are proinflammatory cytokines.^68^ In the presence of HSP60, blocking ERK and JNK did not decrease the proinflammatory profile however, blocking p38 did reduce inflammation.^68^ Thus, a concrete relationship exists between HSP60 and p38 as a downstream modulator in HSP60‐induced inflammation.^68^

Lastly, peptides of HSP60 can also elicit an innate immune response. Peptide fragments of HSP60 have been documented to trigger the innate immune system. To address the fact that *Streptococcus pneumoniae* is poorly immunogenic as other polysaccharides in certain age groups, a study was done to test the ability of p458, a peptide from the sequence of HSP60, to evoke a robust immune response.^73^ Results showed that p458 conjugated to pneumococcal conjugated polysaccharide type 4 activates macrophages to release IL‐12 via a TLR4 activation.^73^

As such, HSP60 exhibits some considerable signaling properties and acts as a potent activator of a wide range of intracellular and extracellular responses in nonimmune as well as innate immune cells as previously mentioned. However, the role of HSP60 in the immune system is much more complex and participates in conjunction with the adaptive immunity system acting as a dynamic link. In the following section, some of the regulatory roles of this protein in the adaptive immunity will be further explained by illustrating the dual role HSP60 possesses as a proinflammatory and anti‐inflammatory molecule.

## 7 HSP60 IN ADAPTIVE IMMUNITY

Innate receptors recognize predictable ligands or antigens and mount an inflammatory response. However, T cell receptors (TCRs) of the adaptive immune response function differently. TCRs collectively recognize various antigens and are clonally distributed, promoting proliferation, and activation of antigen‐specific cells, generating T cells or memory B cells^74^ (Figure 1). The ability of HSP60 to behave as a foreign or self‐antigen and as a danger signal that elicits a strong immunogenic response bestows it properties to participate in the adaptive immunity as well through various mechanisms closely linked with the innate immunity, especially after its interaction with APCs because these cells mediate T cell recognition.^74^ APCs carry antigens to lymph nodes where recognition and priming of naive T cells occur. DCs express costimulatory proteins such as CD80 and CD40 that aid in activation of T cells as well.^74^

In vitro, purified human HSP60 stimulates T cell proliferation in an autoreactive manner, the basis of many autoimmunity and inflammatory diseases, specifically the CD45RA^+^RO^−^ subset.^75^ By stimulating T helper (Th) 1 cells, it promotes cytokine production, mainly IL‐15 and IL‐12 by innate immune cells.^76^ On the other hand, HSP60 peptides can directly stimulate T cells via TLR4 and TLR2 and TCRs to either activate effector T cells promoting inflammation or induce antiergotypic T cells arresting inflammation.^76^ The latter are regulatory T cells (Treg) that recognize peptides which are processed by the MHC molecules of activated effector T cells through TCRs.^76^ HSP60 or its peptides function as an ergotope, an activation marker, and suppresses proinflammatory responses elicited by effector T cells by stimulating anti‐ergotypic T cells in vitro.^76^ It is important to notice that anti‐inflammatory responses can be observed with low concentrations of HSP60: upon interaction with TLR2, Tregs are activated and they elicit CD4^+^CD25^−^ effector T cell suppression, thus inhibiting cytokine production and proliferation.^77^ Activation of Tregs requires about one thousand times less HSP60 than what is needed for TLR4 activation in monocytes and B cells.^77^ Therefore, lower quantities of free HSP60 (the complete protein or certain peptides), induce anti‐inflammatory properties, whereas higher quantities of HSP60 in both forms induce proinflammatory mechanisms.^78^

Earlier studies on autoimmune diseases have explored in depth the anti‐inflammatory properties of HSP60 and its function as an ergotope. T cells recognize HSP60 produced endogenously by cells undergoing stress at the site of inflammation, captured by MHC‐II molecules of APCs.^79^ In a study conducted by Quintana et al.,^80^ efforts were made to identify the regulatory mechanisms in adjuvant arthritis of HSP60 T cells by using DNA vaccination combined with HSP60 fragments. After administration of the vaccine, there was a boost in IL‐10 and transforming growth factor β (TGF‐β) concentrations, immunomodulatory cytokines that ameliorate experimental arthritis, and a decrease in interferon γ (IFN‐γ) secreted by effector T cells by a shift in type 2/3T helper cell (Th2/3).^80^ It seems T cells activated by vaccination with HSP60 peptides are mobilized to the joints affected by arthritis secreting IL‐10 and TGF‐β to finally regulate the T cell groups that potentiate adjuvant arthritis.^80^ In some experimental studies regarding the repressing nature of T cells toward self‐HSP60 in rheumatoid arthritis, it has been demonstrated that recognition of certain self‐epitopes of HSP60 leads to the production of suppressive cytokines by regulatory type 2 cells such as TGF‐β and IL‐4 accompanied by a decreased production of IL‐2 and proliferation in contrast with the recognition of mycobacterial HSP60, where T cells showed greater reactivity and cytokine production.^81^

On the other hand, stimulation of B cell activation and proliferation by HSP60 in mice have been observed in a dose‐dependent manner, also demonstrating additional changes including induction of the synthesis and expression of costimulatory molecules in B cells including CD86, CD40, and CD69. Furthermore, along with the secretion of IL‐10 and IL‐6, immunoglobulin (Ig) G3 was secreted by Ig switch machinery.^82^ Signaling of B cell activation through TLR4 and MyD88 and stimulation of allogeneic T cells in vitro by B cells to produce IFN‐γ are other processes activated by HSP60.^82^ A simultaneous activation of TLRs and B cell receptors by TLR ligands such as HSP60 has been documented, therefore B cell induction for antibody production can also be stimulated by this protein or its peptides.^78^ Apart from these mechanisms where HSP60 acts as a potent activator of select signaling pathways, its role in autoimmunity and the development of some diseases has also been extensively studied. Autoantibodies for self‐HSP60 have been found in inflammatory diseases including rheumatoid arthritis, multiple sclerosis, Bechet's disease, type 1 diabetes, lupus, inflammatory bowel disease and last but not least, atherosclerosis and HF, which will be further discussed in depth.^63^, ^78^, ^80^, ^83^, ^84^

Antigen mimicry, a phenomenon that occurs secondary to high sequence homology between bacterial and mammalian HSPs also elicits strong autoimmune responses, creating a correlation between bacterial infection and an ensuing autoimmune response. It has been observed in periodontitis that bacteria representative of this disease such as *P. gingivalis*, *Aggregatibacter actinomycetemcomitans*, and *Bacteroides forsythus* express HSP60 homologous with *E. coli*, GroEL, and periodontitis patients tend to have serum antibodies to the bacterial HSP60.^85^ In a study conducted in healthy patients and diabetes mellitus type 1 patients, a robust immunoreactivity was observed toward peptide 19 (Pep19), derived from HSP60 expressed by *P. gingivalis* (PgHSP60) showing a “epitope spreading” pattern, the unfurling of an autoimmune response specific to an antigen to different epitopes, in both groups.^86^

From this preamble, we get a good grasp of the repertoire of functions HSP60 features in the immune and nonimmune realm as well as the robust capacity it has to elicit an inflammatory response, or even mitigate it, as observed in some of the studies previously mentioned. All of these tasks combined give rise to the potential capacity of HSP60 or its peptides to participate at different stages of CVD progression, which can be at early atherogenesis or in the final stages of a failing heart. In the following section, we will review some of the recent investigations done on HSP60 in the spectrum of CVDs, highlighting the relevant findings and its implications for therapeutic purposes.

## 8 HSP60 IN THE DEVELOPMENT OF CVDS

CVDs are the consequence of a plethora of stress‐inducing stimuli that may act upon cardiac tissues, mainly the myocardium, disabling the heart's structure and function. Coronary artery plaques are major contributors of progressive decline in delivery of oxygen and nutrients to the myocardium. In situ inflammation is also an important feature of ongoing injurious events, and can manifest as cytokine secretion from resident cells, triggering proinflammatory programs and recruiting cells of the immune system to affected areas. Failure to clear the initiating stressor results in repeated injury to the tissues involved and induction of programmed cell death, a common finding leading to organ dysfunction in these pathologies.^87^ mtHSP60, a cytoprotective protein and ally relevant for normal cell functioning, seems to participate in the pathogenesis of different CVDs. Literature has indicated that extracellular locations of the chaperonin contribute to the unraveling of various detrimental events leading to progression of CVDs contrasting the normally attributed functions of proteostasis.^88^ These results have paved the way for exploring therapeutic strategies in different modalities and is a field in under constant evolution.

### 8.1 Hypertension

High blood pressure remains as one of the leading causes of mortality worldwide, estimates going as high as 10.4 millions of deaths per year. The global burden remains elevated and its impact in cardiovascular morbidity and mortality still remains as an important contributor and risk factor despite efforts by different committees to tackle and treat patients before falling in the category of hypertension.^89^ Every year, evidence‐based guidelines, objectives, and recommendations are published, to guide clinicians to treat patients with appropriate measures, as the prevalence of raised blood pressure is increasing globally and the outcomes of this disease impact cardiovascular health negatively giving rise to the development of complications in the near future.^89^ HSPs, throughout years of research, have been intimately associated with vascular disease however its role in hypertension specifically, is not well established. Some studies that have shed light on its involvement in hypertension will be discussed in this section.

It has long been reported that patients with hypertension manifest clinical improvement after taking dry sauna baths, although the exact factors related to this observed amelioration remain partially unknown.^90^, ^91^ In an in vivo model using Dahl salt‐sensitive hypertensive rats which developed hypertension such as a result of a high dietary salt intake, Oyama et al.^92^ studied the effect of repetitive hyperthermia in the form of short‐duration hot water baths as a mean to reduce systemic blood pressure, cardiac remodeling, and mechanical function in hypertension‐induced cardiac hypertrophy. HSPs, including HSP90, HSP70, and HSP60 were evaluated in left ventricular (LV) tissue samples, since their overexpression responds to hyperthermic stimuli.^92^ Interestingly enough, cardiac HSP60 levels are decreased in rats fed with a high salt diet, while normal salt diets lead to a higher HSP60 expression.^92^ Repetitive hyperthermia resulted in an overall increase in all HSPs assessed, and it was further determined that high salt diet group rats subjected to repetitive hyperthermia exhibited clinical improvement compared to high salt diet only animals as manifested by decreased afterload, myocardial oxidative stress, and inflammation, while successfully preserving telomerase activity.^92^ These results shed some light on the role of HSPs’ activity on preventing hypertension‐related tissue damage by means of experimental repetitive hyperthermia.

A comparative study was done to determine the differences in serum concentrations of anti‐HSP60, HSP60 and antimycobacterial 65‐kDa protein antibodies between normotensive and borderline hypertensive individuals.^93^ Markedly higher concentrations of HSP60 were identified in patients with borderline hypertension, and this was associated with higher intima/media (I/M) thickness of the carotid arteries, indicating possible plaque presence, whereas levels of anti‐HSP60 where slightly lower in the same group of patients.^93^ This is the first report that establishes the relationship between borderline hypertension and HSPs, highlighting especially the presence of HSP60.^93^ This study contrasts in some aspects with another study conducted by Zhang et al.^94^ where efforts were made to demonstrate a risk‐associated presence of anti‐HSP60, hypertension and diabetes for coronary artery disease (CAD) in a Chinese population. Higher levels of anti‐HSP60 were found in subjects with hypertension versus those without.^94^ Also, higher levels of anti‐HSP60 were linked with a higher risk for CAD in a dose‐dependent manner.^94^ When evaluating the combined effects of hypertension, anti‐HSP60 and diabetes for CAD, higher levels of anti‐HSP60 jointly with the presence of hypertension were linked with a fourfold increased risk for CAD versus normotensive subjects. Similarly, diabetic and hypertensive subjects with higher levels of anti‐HSP60 presented with more than 20‐fold risk for CAD.^94^ As it is a well‐known fact that the etiologies of CAD include diabetes, hypertension and atherosclerosis and in the latter, a higher presence of HSP60 and HSP60‐specific T cells can be found, the data of this study in CAD patients is consistent with those findings. To have a better understanding of the role of HSP60 in these cardiac diseases, studies pertinent to the presence of HSP60 and its relationship to atherosclerosis will be discussed in the following segment of this review.

### 8.2 Atherosclerosis

Atherosclerosis is a serious disease with a slow but steady progression that occurs due to a number of chronic inflammatory processes in the arterial intima, a layer of the arterial wall just below the endothelium, which when amplified by different factors, can cause partial or complete obstruction of the vessel leading to a life threatening insult to the heart. Now considered an autoimmune disease, the chronic inflammation generally culminates in the formation of a plaque which menacingly narrows the vessel's diameter, depriving the heart muscle of adequate blood flow and sufficient oxygen causing cardiac ischemia. However, if a sudden rupture of the plaque occurs, coronary artery occlusion can occur, causing a myocardial infarction.^87^ Evidence has demonstrated that bacterial and human HSP60 participate in the pathophysiology of atherosclerosis at different points of the disease progression elucidating its importance as a marker of disease and as a potential target for treatment.^9^, ^10^, ^95^, ^96^, ^97^

A study reported by Xu et al.^95^ might be one of the first papers where efforts were made to establish the role of HSPs in atherogenesis. Normocholesterolemic rabbits were immunized with HSP65, a major antigenic component of *M. tuberculosis* (mHSP65). Rabbits under a cholesterol‐rich diet who were immunized with HSP65 as well developed a more serious degree of atherosclerosis than those only immunized or fed with a cholesterol‐rich diet alone.^95^ Such studies utilizing experimental immunization with different antigens, in this case HSPs, have revealed mechanisms in atherosclerosis where immunity cells, antigen‐antibody deposits and chronic inflammation are important culprits for disease induction and progression. Supporting this evidence, a study conducted in a group of patients revealed that anti‐HSP65 antibody levels in those with atherosclerosis were significantly higher compared with those without the disease establishing initially an interesting link that played a significant role in future studies about atherogenesis which will be further discussed.^98^

As an immunogenic antigen, after infection or vaccination the subject develops immunity against bacterial HSP60.^99^ It has been hypothesized that an autoimmune response to human HSP60 could be a central mechanism for the pathogenesis of atherosclerosis. Between human HSP60 and bacterial HSP60 [which includes the mycobacterial homologue (mHSP65), *C. trachomatis* HSP60, and the *E. coli* homologue GroEL] cross‐reactivity occurs due to a 95% sequence homology between bacterial HSPs and a 50%–55% sequence homology between bacterial and human HSP60.^63^, ^99^ This phenomenon has been well established in patients with periodontitis as multiple studies have indicated that patients with chronic periodontitis have a greater risk of presenting coronary artery disease.^85^, ^100^, ^101^, ^102^ Underlying immune mechanisms against periodontal pathogens, specifically PgHSP60, have been associated with atherosclerosis.^103^ This is evidenced by markedly higher levels of antibodies to PgHSP60 and human HSP60 in subjects with atherosclerosis compared to the groups of healthy subjects and subjects with periodontitis alone.^103^ Also, antigen‐specific T cells to PgHSP60 and human HSP60 have also been detected in atherosclerotic tissues and in circulation indicating that HSP60 expressed under stress conditions on endothelial cells can interact with anti‐PgHSP60.^103^ Pep19, a peptide from PgHSP60 also has the capacity to stimulate low‐density lipoprotein (LDL) oxidation, a risk factor for the progression of atherosclerosis.^96^ Therefore, interaction of bacterial HSP60 and self‐HSP60 with the immune system confers a greater risk for atherosclerosis development through immune recognition and reaction by circulating innate and adaptive immunity cells as homology between these two counterparts has been greatly preserved throughout the years.^104^ Atherosclerosis is perhaps the CVD with the greatest involvement of inflammatory disorders, and HSP60 homology and identity between species, exerts and enhances an autoimmune component in this pathology. This process is initiated by endothelial cells subjected to stress which express surface HSP60 triggering an anti‐HSP60 immune response prior established by previous exposure to bacterial HSP60.^104^ Thus, understanding the recognition mechanisms of HSP60 in the body, during infection or new vaccination strategies with foreign HSP60 can greatly influence the outcome of some inflammatory mechanisms of atherosclerosis.

Regarding the presence of endogenous HSP60 in the circulation, some studies have linked possible mechanisms of disease progression in atherosclerosis to this phenomenon, including cell death due to stress, inflammation or infectious agents as previously described, and soluble HSP60 (sHSP60) locally released from atheromas.^105^ The protein sHSP60 has cytokine‐like activity and triggers the expression of TNF‐α from macrophages, while stimulating E‐selectin expression, vascular cell adhesion molecule‐1 (VCAM‐1), and intercellular adhesion molecule‐1 (ICAM‐1) by endothelial cells, which serve as markers for endothelial cells activation.^105^ Higher anti‐human HSP60 antibody levels in individuals with a high cardiovascular risk in comparison with healthy individuals has also been reported, as well as higher sHSP60 concentrations in patients with prevalent/incident carotid plaques.^105^, ^106^ With all gathered data, the link between the presence of anti‐HSP60 antibodies and sHSP60 and the development of atherosclerosis is evident, elevating the importance of these proteins as prognostic biomarkers for risk.^105^ Similar to the immune recognition of exogenous HSP60 and sHSP60 an immune response solely against translocated HSP60 can also be mounted under pathological conditions. Patients subjected to vascular stress responses by atherosclerosis risk factors demonstrate self‐HSP60 translocation to the cell surface in endothelial cells where it behaves as a stress signal recognized by a variety of immune cells circulating in the periphery.^107^, ^108^ Similar to sHSP60 release, intracellular stress seems to be the determinant factor tied to HSP60 release into the cytosol, from where it may then translocate to the lipid bilayer acting as a DAMP for innate and adaptive immunity interaction.^9^

Other mechanisms attributed as possible contributors to atherosuceptibility include vascular shear stress and heterogeneity in endothelial phenotypes.^109^, ^110^ It has been proposed repeatedly that atherosclerosis develops by an initial infiltration of immune cells in the intima, classically in certain sites where predilection is higher than other places such as arterial branches and curves.^111^ Several antigens have been documented to trigger adaptive and innate responses in atherosclerosis to induce the initial immune cell infiltration, however the top three triggers for activating specifically T cell mediated immune responses are apolipoprotein B‐100 (ApoB‐100), oxidized LDL (oxLDL), and HSP60/65.^111^ Thus, the expression of these molecules influenced by various hemodynamic factors and vascular damage that characterize atherosclerosis could become one of the central precipitating factors to initiate atherogenesis and perpetuate a vicious cycle of chronic inflammation.

A recent study compared the activating effect that oxLDL and HSP60 have on T cells via DCs and whether these two antigens depend on each other for activation.^112^ DCs were treated with human serum albumin (HSA) conjugated with malondialdehyde (MDA); posteriorly, autologous T cells obtained from atherosclerotic lesions were cultured with these pretreated DCs.^112^ MDA‐HSA elicited inflammation via DCs‐mediated T cell activation and by direct T cell activation, processes that were inhibited by antibodies against MDA.^112^ HSP60 was also strongly recognized by T cells activated by MDA‐HSA.^112^ They hypothesized that oxLDL promotes inflammation by indirectly promoting recognition of HSP60 by macrophages, a theory that was consequently proved to be correct as silencing of HSP60 suppressed DCs‐mediated oxLDL‐induced T cell activation through DCs.^112^ T cell activation requires recognition of HSP60 epitopes, for which presentation of immunogenic peptides via MHC molecules is necessary. Analysis of carotid endarterectomy samples from patients with carotid artery stenosis identified on the surface of macrophages the presence of HSP60 on both *vasa vasorum* and carotid artery endothelial cells using immunohistochemistry.^97^ Serum titers for antibodies for HSP60, *C. pneumoniae* and cytomegalovirus were increased, although no markers related to infection to these last two pathogens were found.^97^ These findings support the idea that infection may be one of the initiating factors for atherosclerosis, where high sequence homology and epitope sharing between self‐HSP60 and its microbial counterparts may promote antibody secretion with subsequent deposition of immune complexes and endothelial dysfunction.

As HSP60's implicated pathway in atherosclerosis includes activation of T cells, monocytes, and DCs, it facilitates the attachment to endothelial cells and transmigration into the intima as demonstrated in a study that reported T cell activation by HSP60 through DCs in a MHC‐II dependent‐fashion, when DCs were cocultured with HSP60 and T cells.^113^ Results demonstrated a strong production of HSP60 antigen‐specific T cells as determined by CD25 expression in this population.^113^ HSP60 also induced DCs maturation (mDCs) and the mDC‐T cell activation elicited type 1/17T helper cell (Th1/17) cytokine production from healthy patients and patients with possible CVDs.^113^ In the same study, a plasma protein with antithrombotic properties, called ANXA5, inhibited HSP60‐mediated T‐cell and mDCs activation, possibly dampening immune responses elicited by HSP60 by weak binding to the protein.^113^ The antigen presentation can be performed both by APCs and by endothelial cells and VSMCs expressing MHC, both class I and class II. T cells have receptors γδ or αβ. Those that express αβ can have either CD4, (coreceptor to MHC‐II) or CD8, (coreceptor to MHC‐I). CD4‐T cells are sub categorized as Th17, Th2 and Th1, Treg and TFH cells. Th1 cells have been predominantly present in atherosclerotic lesions of mice and human, and are implicated in macrophage activation, and release of IFN‐γ and IL‐2 cytokines, playing an important part in the pathophysiology of atherosclerosis.^114^, ^115^ After infiltration of lymphocytes, monocytes and DCs into the subendothelial space, interaction between anti‐HSP60 antibodies and surface HSP60 leads to significant cell damage, a phenomenon that occurs particularly under stress conditions, paving the way to atherogenesis.^116^

Activated T cells in atherosclerosis are an essential component in atherosclerosis. In atherosclerosis‐prone (LDLR^−/−^) and lymphocyte‐deficient (RAG1^−/−^) mice, atherosclerotic lesions development was reduced by 54% in comparison with only atherosclerosis‐prone (LDLR^−/−^) mice.^117^ A link has been established between the concentration levels of HSPs and the severity of atherosclerosis, reporting a localized enrichment of γ/δ T cells in atheromatous lesions.^111^ Among the various subsets of T cells, mostly CD4^+^ are the first to extravasate.^9^ Concentrations of HSP60 antigen‐specific T cells apparently are higher in young healthy patients and are linked to increased intima‐media thickness at different vascular territories in comparison with levels of anti‐HSP60 which do not link to increased intima‐media thickness in elderly patients proposing that HSP60 takes on an important task at the initial stages of atherosclerosis.^83^ It has also been suggested that before the initial antigen‐driven T cell lesion, an appropriate microenvironment must ensure, created through the presence of CD4^+^ and CD8^+^ T cells as well as DCs and macrophages.^83^ In endothelial lesions, T cells populations consist predominantly of CD4^+^ memory effector cells.^83^ Furthermore, HSP60 leads to activation of specific CD4^+^CD25^+^CD45RO^+^ T cells, which engage with endothelial cells that express HSP60, forming adhesion molecules (VCAM‐1 and E‐selectin) at sites with predisposition for progressive development of atherosclerotic lesions after exposure.^83^

Thus, several intricate mechanisms participate in atherosclerosis via the innate and adaptive immunity and HSP60 has proven to be a potentially strong antigen that elicits a cascade of inflammatory processes making the site of the atherosclerotic plaque a center of chronic inflammation and an autoimmune target. Once HSP60 is localized in the vicinity, it can cause harmful endothelial injury and plaque development, acting as a powerful signaling protein, making it an essential component of the pathophysiology of atherosclerosis.

### 8.3 Coronary artery disease and AMI

HSP60 is well known to be intimately related to the initiation and progression of atherosclerosis, and it is thus considered a risk factor for it. However, there is vast evidence of HSP60's involvement in further stages of cardiac disease progression as well. The rupture of an atherosclerotic lesion and the consequential stoppage of coronary artery blood supply to the myocardium due to vessel‐narrowing or blockage collectively leads to CAD or ischemic heart disease, a pathology that carries a high mortality rate.^118^ After repetitive or extensive myocardial damage (myocardial infarction), HF occurs inevitably, and the outcome becomes poor.^119^ AMI is the primary cause of high cardiovascular mortality and morbidity that occurs worldwide. In this setting, chronic myocardial ischemia due to coronary blood flow obstruction, a dangerous consequence of atherosclerosis or plaque rupture, or acute lack of perfusion, as manifested during cardiogenic shock, deprives muscle tissue from oxygen and nutrients, which in turn alters tissue homeostasis and induces cell metabolic reprogramming and cell death. Shortage of intracellular ATP and ROS generation in response to hypoxia are known to be important mechanisms in myocardial death, however they are not the only participants.^120^ In this section, we will discuss the role of HSP60 in CAD and myocardial infarction as well as recent findings of its involvement in the pathophysiology of these diseases.

In the human heart, the role that HSP60 plays in the setting of CAD has been long proposed by Knowlton and Strivatsa,^121^ however a greater body of evidence was necessary to support these findings. For this purpose, a large case‐control study was conducted in a Chinese cohort by Zhang et al.^94^ in which they determined the relation between HSP60 and anti‐HSP60 in CAD. Results demonstrated an increase in serum HSP60 and anti‐HSP60 in CAD patients, and when taken together, were linked to a twofold risk for the disease.^94^ Another study identified increased concentration levels of salivary IgA to different oxidized epitopes, which act as DAMPs recognized by PRRs, as previously mentioned, in different pathological circumstances.^122^ They identified higher levels of salivary IgA in CAD and atherosclerotic patients to MDA acetaldehyde–modified LDL and epitopes of pathogens such as gingipain A hemagglutinin domain of *P. gingivalis* (Rgp44), and *A. actinomycetemcomitans* HSP60 (AaHSP60) in comparison to non‐CAD patients reinforcing the importance of the role of bacterial HSP60, specifically oral pathogens, as risk factors for the progression of CVD.^122^

High levels of HSP60 in serum have been identified in multiple settings in CAD. In C57BL/6J mice that underwent coronary artery ligation a rapid rise of phosphorylated IRAK‐1 (TLR4‐MyD88 signaling pathway) and HSP60 intracellular depletion were seen owing to increased secretion into the extracellular space, with HSP60 ability to activate extrinsic apoptotic pathways in cardiomyocytes, via caspase‐8, perpetuating deleterious effects in myocardial ischemia.^123^ A prospective clinical study including asymptomatic patients from the Multi‐Ethnic Study of Atherosclerosis‐MESA, identified that both IL‐2 and anti‐HSP60 serum levels correlate with the coronary artery calcification score, even after adjustment to classical factors from the Framingham coronary heart disease risk score.^124^ Coronary artery calcification score is used to assess underlying asymptomatic atherosclerosis and is a predictor of future cardiovascular events in the general population.^124^ An autoimmune link could explain why both serum markers may be associated with CAD. IL‐2 stimulates T cell proliferation and is secreted by naive CD4^+^ T cells and proinflammatory Th1 cells. On the other hand, HSP60 serum protein levels correlate with its serum antibodies.^124^ Both cellular and humoral inflammation induced by HSP60 may contribute to increased coronary artery calcification scores.^124^

A study aimed toward the patterns of expression of monocytes in patients with unstable angina, a disease that is part of spectrum of CAD, reported that HSP60 is expressed under stress conditions where correct blood flow is hindered to the myocardial tissue, and induces IL‐12p70 secretion by intermediate monocytes, a subset involved in antigen presentation.^125^ IL‐12p70 subsequently induces Th1 lymphocyte differentiation, which in turn produces high levels of IFN‐γ, promoting activation of macrophages, endothelial cells and VSMCs, rendering instability to the plaque and facilitating rupture.^126^ Specifically, CD14^++^CD16^+^ subset of intermediate monocytes stimulated by HSP60 demonstrated higher levels of chemokine expression, such as C‐C chemokine receptor type 2 (CCR2), CCR5, and CX3CR1.^125^ These monocytes also exhibited higher expression of PRRs such as TLR2, TLR4, and CD36.^125^ TLRs have a primordial role in the progression of atherosclerosis as they participate in the recognition of oxLDL and HSP60, and the production of inflammatory cytokines as previously described.^3^, ^52^, ^69^ Hence, an enhanced proinflammatory profile was observed in such patients, contributing to progression to AMI. In a post‐AMI setting, HSP60 levels seem to positively correlate with other cardiac enzymes, such as troponin and CK‐MB, which can be attributed to release of these proteins by necrotic cardiomyocytes into the bloodstream allocating the chaperonin a potential prognostic value.^127^

Apart from Th1 polarization that occurs in the active phase of CAD, it is crucial to emphasize the fact that Th1, Th2, Th17, and Tregs subsets have already been identified in atherosclerotic plaques^128^, ^129^, ^130^ and numerous studies have delineated specific inflammatory cellular groups that participate in atherosclerosis. However, further research is needed to identify and observe the behavior of precise inflammatory cell subsets at different stages of CAD. Regarding the expression levels of Th17 cells and monocytes, patients with stable and unstable angina, ST‐elevated myocardial infarction and non‐ST elevation myocardial infarction, demonstrated a gradual increase by an uncontrolled activation of these cell population due to a poor regulation by Tregs.^131^ Higher expression levels of IL‐23/Th17 related genes were also observed accompanied by an elevated proportion of CCR2 positive monocytes compared to the control group.^131^ Moreover, CCR2 positive monocytes promote IL‐23 induced Th17 cell‐expansion and produce IL‐6.^131^ Thus, HSP60 might be responsible for this induction due to Th17 being directly responsive and undergoing immediate expansion by HSP60 in a previous study.^132^

In this regard, the innate and adaptive immune response to locally released endogenous cellular molecules such as HSPs, fibrinogen and soluble heparan sulfate, which occurs after ischemia, has a primordial role in aiding tissue inflammation and damage through cytokine secretion and leukocyte recruitment. Thus, these components together carry heavy weight as important contributors to the overall inflammatory processes that occur.

### 8.4 Arrhythmias

Arrhythmia is one of a kind in terms of pathophysiology understanding and curative methods. During recent years many new therapeutic options to control, prevent or cure arrhythmogenic hearts have been developed. Novel focuses are currently on therapeutic options; some in the form of pills, others in interventional methods, but it is remarkable that a complete understanding of arrhythmogenesis is still missing. Currently it can be named only some of the risk factors for developing sudden (and lethal) or chronic arrhythmias, such as hypertension, atherosclerosis, obesity, some metabolic states, certain drugs or increasing age; but it would be great to recognize arrhythmia‐prone hearts with molecular markers.^133^

Chronic atrial fibrillation (CAF), the most common arrhythmia in adult patients, is the most studied rhythm. Recent lines of investigation regarding molecular expression patterns, including some DAMPS such as HSPs, have focused on myocardial adaptation responses to chronic or acute insults while having CAF. In a study performed by Schäfler et al.,^134^ atrial samples were obtained from 14 patients who underwent elective cardiovascular surgery. Eight out of these patients were previously diagnosed with CAF and the remaining six had sinus rhythm.^134^ The group reported a 2.5‐fold rise in HSP60 levels within the myocardium of CAF patients compared to those with sinus rhythm, which led to the hypothesis that the chaperonin could be related to the pathophysiologic processes in arrhythmogenesis.^134^ Since the protein‐folding activity of HSP60 depends on its association with HSP10 to form a functional HSP60/HSP10 complex, Schäfler et al.^134^ further set to determine the expression HSP10 in atrial myocardium from patients with CAF. Consistent with their previous results, a 2.3‐fold rise in HSP10 levels was observed in CAF myocardial samples compared to sinus rhythm controls, while HSP60 saw a 2.4‐fold increase in CAF in affected patients.^134^ The simultaneous expression increment observed for these two HSPs may serve as an adaptive response to the increased energy demands due to chronic fibrillating stress.

HSPs have been studied in patients with permanent CAF after mitral valve surgery, and its capacity in stabilizing spontaneously restored sinus rhythm. A group of 135 patients who were previously diagnosed to have permanent CAF (for a year or more before surgical intervention) were more over separated into two groups, a sinus rhythm group and an atrial fibrillation group, on the basis of recurrence of atrial fibrillation or persistence of sinus rhythm after the next 7 days following surgical intervention.^135^ Atrial samples from these groups revealed lower HSP60 protein levels in patients with restored sinus rhythm compared to those from the atrial fibrillation group. Furthermore, less myocyte apoptosis and tissue myolysis in the sinus rhythm group was also observed.^135^ Likewise, venous blood samples were used to determine the proinflammatory cytokine levels such as TNF‐α and IL‐6, and the results showed an increase in the atrial fibrillation group.^135^ Overall, higher levels of atrial HSP60 were linked with higher risk for the recurrence of atrial fibrillation after mitral valve replacement, postulating this intracellular chaperone as a feasible biomarker for determining the outcome of patients after surgery.^135^

Recently, the effects of inflammatory biomarkers for predicting recurrent atrial fibrillation following ablation therapy have been studied.^136^ Some of the possible implications of several molecules including DAMPs, HSPs, and cytokines in relation to recurrent atrial fibrillation were described.^136^ HSPs are recognized to serve in a bimodal fashion, attributed to the degree of myocyte damage. Authors describe two different models of action in which specific intracellular chaperone actions of HSPs (including HSP27, HSP60, and HSP70) move balance toward inhibition of atrial remodeling; and extracellular inflammatory actions of HSPs (when damage to myocyte is severe) trend toward atrial remodeling.^136^ Intracellular actions of HSPs have been shown to moderate protein stabilization and refolding versus protein degradation on less damaged proteins, and to activate HSF‐1 which ends degrading the more severely damaged proteins.^136^ HSPs also interact with calcium homeostasis, cytoskeleton and ion channels.^136^ Nonetheless, the exact mechanisms of HSPs' appearance in serum of patients with insults during CAF are still debated and need further investigation. From these proteins HSP27 has proven to be the most likely to correlate with recurrent atrial fibrillation prognosis.^136^ Findings support that high levels of HSP27 are related to lower levels of remodeling with decreased progression to recurrent atrial fibrillation by the following mechanisms: HSP27 stabilizes the cytoskeleton by bonding to F‐actin and ɑ actin; it helps myocyte membrane potential maintenance by binding to l‐type calcium channels; it inhibits TNF‐α pathways and increase IL‐10, an anti‐inflammatory cytokine.^136^ As for HSP60, its power for predictability of recurrent atrial fibrillation is well defined for other procedures such as mitral valve replacement; with measurement of intracellular and serum HSP60 levels.^136^ Nonetheless, information in regard to its role in recurrent atrial fibrillation after other insults is controversial.^136^

To establish the participation of HSPs on the progression and different stages of atrial fibrillation, the presence of some HSPs, including HSP73, HSP72, HSP27, and HSP60 in sinus rhythm controls and in patients with persistent atrial fibrillation or paroxysmal, were studied.^137^ No statistically significant difference was identified between these proteins in any of the three conditions.^137^ However, correlation analysis of HSPs indicated a positive association between HSP60 and HSP72 in sinus rhythm patients not observed otherwise in atrial fibrillation, while in this last group, a negative association between HSP73 and HSP27 was noticed.^137^ Moreover, in determining the degree of myolysis, a consistent feature in atrial fibrillation, HSP60 was found to be significantly decreased in moderate, severe and profound myolysis states compared to the slight myolysis state.^137^ It is possible that the loss of cytoprotective effects ascribed to HSP60, as seen hereby in the advanced stages of atrial fibrillation, may account for higher susceptibility to cellular injury and muscle tissue death.

### 8.5 Heart failure

HF accounts for great morbidity and mortality worldwide and due to its variable etiologies and association with multiple chronic diseases it has become one of the most studied cardiac conditions. The advent of better prognosis from treatment of acute HF events and an ageing population has ultimately led to an exponential increase in prevalence; justifying a need for better understanding of pathophysiology and therapeutic options. In regard to HSPs several studies have been performed in the last few years, generally showing correlation between HF and rising HSP levels.

It has been well established that increased levels of HSP60 are found in failing hearts. Knowlton et al.^138^ conducted an experiment studying the expression of HSPs in HF due to dilated cardiomyopathy (DCM) and ischemic heart disease. The results showed that in DCM hearts, both HSP60 and HSP27 expression increased significantly, 2.5‐ and 2.0‐times, respectively.^138^ However, in ischemic heart disease hearts, HSP27 levels increased but did not differ from normal hearts, while HSP60 showed twice as much of an increase with respect to normal hearts.^138^ In another study with rats with HF, an increase in HSP60 levels of approximately 140%, 8 weeks after coronary artery ligation was observed.^138^ It poses the observed increase in HSP60 as a response to the impairment in oxygen consumption and the decrease in high‐energy phosphates that take place during HF in the mitochondria of cardiomyocytes.^139^ A later study by the same research team, stated that the compensatory stage of HF takes place 1 week after the coronary artery ligation, while the final stage takes place 8 weeks after the procedure, at which times HSP60 expression levels were studied, showing an increase with a similar time pattern to that of their previous study.^140^

Most studies report a similar trend on HSP60 levels during the development of HF. For instance, De Souza et al.^141^ performed a proteomic analysis of the molecular mechanisms that underlie the atrial structural remodeling that takes place during congestive HF. Their results showed a 1.3‐fold increase in HSP60 and HSP27 levels 24 h into the development of the condition, while other proteins, such as α‐B‐crystallin and HSP90, did not show a similar increase until 2 weeks later.^141^ These results draw special interest regarding the behavior of HSP60 levels during HF, suggesting an expression pattern independent from other proteins.^141^ Although the HSP60 expression was parallel to that of HSP27, both in fold‐change and in time, this is not always the case.^141^ Regarding the relation between HSP60 and other HSPs, there seems to be no positive link between the concentration levels of HSP60 and the rest of the chaperone families, including HSP27, HSP70, and HSP90.^142^ In said study, changes in HSP60 and CYP2E1 expression in DCM at the end stage of HF were studied.^142^ Their results indicate that protein accumulation in the mitochondria, resulting from an alteration in oxidative phosphorylation cycles, may be a source of stress to trigger HSP60 expression.^142^

Interestingly in HF, HSP60 follows an abnormal distribution. In a study, 66% of the total HSP60 was found in the mitochondria, 25% in the cytosol, and the remaining 9% was located in the plasma membrane.^17^ The same study suggests that HSP60 is associated with apoptosis when found in the plasma membrane specifically, this was concluded by means of isolating cardiomyocytes from failing hearts and testing for activated caspase‐8, an indicator of the extrinsic pathway of apoptosis.^17^ On the contrary, HSP60 showed antiapoptotic and protective behavior when in the mitochondria or cytosol.^17^ Adding up to these findings, Kim et al. demonstrated that HSP60 levels are doubled in HF, and concurred in that the chaperonin is present embedded in the plasma membrane; furthermore, they mention that it is also found extracellularly, where it can activate apoptotic pathways on cardiomyocytes via TLR4 activation as previously described.^69^

The potential predictive role of HSP60 in assessing HF severity and outcome has also been studied.^143^ HSP60, glomerular filtration rate and B‐type natriuretic peptide (BNP), seem to be independent indicators that anticipate the possible effects in patients with congestive HF.^143^ It was also observed that patients with increased levels of circulating HSP60 possessed a greater risk of cardiac episodes and morbidity, showing hyponatremia and renal dysfunction as well.^143^ In contrast, in a study by Buriro et al.,^144^ aimed at studying acute HF induced by heat stress, the levels of HSP60 and HSF‐1 were evaluated by means of subjecting myocardial cells of neonatal rats to high temperatures in vitro. Their results showed no link between mitochondrial RNA (mRNA) levels of HSP60 and HSF‐1 and their respective proteins.^144^ It was also observed that HSF‐1 is not the sole modulator of HSP60 expression, due to nonconsistent patterns when comparing their expression.^144^ Thus, the importance of HSP60 as a predictor of severity in HF might vary depending on the etiology.

Acute fluoride (F^−^) toxicity is yet another known event related to acute HF, where cardiovascular impairment is manifested by electrolyte imbalances leading to ventricular arrhythmias, a strong oxidative response with concomitant decrease in its antioxidative counterpart, induction of myocardial apoptosis and necrosis, ATP depletion and cytoskeletal dysfunction. Since HSP expression responds to all of the aforementioned stress‐inducing stimuli, Panneerselvam et al.^145^ characterized the cardiac expression profile of some of the members of this family of proteins, including HSP27, HSP32, HSP40, HSP60, HSP70, and HSP90, as well as the HSF‐1 transcription factor, in an in vivo rat model of acute Fl^−^ toxicity. A dose‐dependent increase in both myocardial transcript and protein levels for HSPs and HSF‐1 including HSP70, HSP60, HSP32, and HSP27 was reported, while an inverse pattern was observed for HSP40 and HSP90.^145^ In this model, HSP60 overexpression is suggested to result from increased apoptotic and oxidative events induced by acute Fl^−^ toxicity.^145^

Chronic hypersympathetic activity is a frequent finding in HF as indicated by high resting heart rate (HR). In an organ with an already compromised contractility, persistent stimulation by the sympathetic nervous system (SNS) implies increasing the workload the heart is subjected to, and the resulting stress this system is endowed with activates endogenous defense mechanisms, including the HSPs response. In this context, Afanasiev et al.,^146^ studied the therapeutic potential of transcutaneous electrical stimulation of the auricular branch of the vagus nerve, as a means of stabilizing basal HR in *New York Heart Association* Functional Class (FC) III and IV HF patients. Moreover, based on literature reports suggesting that vagus nerve stimulation leads to an increase in HSP70, they also sought for a causal role of HSP60 and HSP70 as cellular resistance factors induced by the therapeutic intervention.^146^ Their results showed that transcutaneous electrical stimulation was associated with an overall clinical improvement in 58 out of 63 participants, from which 3 FC IV patients moved to FC III, 52 moved from FC III to FC II, and 3 went from FC III to FC I.^146^ From further division of the patients who responded to treatment into subgroups according to their resting HR, it was observed that individuals with resting HR ≤ 80 at baseline had an increase in both HSP70 and HSP60, whereas those with HR > 80 only saw elevations for the former.^146^ This study poses HSPs as stress related proteins that may account for the therapeutic effects of transcutaneous electrical stimulation of the auricular branch of the vagus nerve, where individuals from early HF stages (FC I and II) may respond by upregulating members of the HSP family, and that metabolic exhaustion present in hearts from FC III and IV patients may explain the lack of HSP60 activity.^146^

And finally, supporting the well‐established phenomenon that HSP60 binds to TLR4 as described earlier,^52^, ^82^ a study showed that TLR4 in cardiomyocytes could aggravate HF by engaging in inflammatory processes in cases of long‐term myocardial infarction.^147^ As results demonstrate that TLR4 has a high affinity for HSP60 and thus can be activated by HSP60 during HF, the role of HSP60 as a signaling molecule was reinforced which is yet another and vital way the chaperonin relates to inflammation in this pathology.^147^

### 8.6 Idiopathic LV heart dysfunction

Among HF patients, there is a particular subset who display some of the hallmark features of the disease, including increased LV end diastolic diameter and reduced LV ejection fraction, but do not otherwise stem from CAD. Nevertheless, it has been observed that these patients show evidence of coronary microvascular impairment, which is believed to be a result of nonconventional risk factors. Because of their association with CVD and endothelial dysfunction, Giannessi et al.^148^ studied the participation of HSPs against the background of idiopathic LV heart dysfunction, as possible biomarkers related to this disease. Their results demonstrated an increase in serum HSP60, HSP72, and anti‐HSP60 antibodies in patients with idiopathic LV heart dysfunction, suggesting that this finding might be associated to disease severity, with HSP60 being higher in patients with a reported LV ejection fraction ≤ 50%, and HSP72 in more severely affected cases reporting LV ejection fraction < 35%; in addition, the latter group also showed increased serum IL‐6 and CRP.^148^ What is more, IL‐6, anti‐HSP60, and HSP72 correlated significantly with BNP, a major marker in determining HF stages, suggesting that these could serve as biomarkers to assess the degree of ventricular dysfunction in patients without CAD.^148^

### 8.7 Cardiomyopathy

Cardiomyopathy comprises a heterogeneous group of diseases with mechanical or electrical disturbances of myocardium. Etiologies are multiple and the end result is ventricular dysfunction and progressive HF. Association with some types of cardiomyopathies and inflammatory mechanisms has been vastly described in the last few decades.

In relation to DCM a research study demonstrated that the myocardium from DCM patients expresses significantly more HSP60 compared to that from heart donors without CVD.^149^ This was one of the initial hints of the relation between HSP60 and chronic HF states. A fivefold increase via reverse transcriptase polymerase chain reaction (RT‐PCR) in protein levels of the chaperonin has also been demonstrated in the myocardium of DCM subjects, with mRNA levels showing consistent increases in these samples.^149^ In a similar study evaluating HSP60 within DCM affected hearts, an increase in levels of mtHSP60 levels with a simultaneous rise in human cytochrome P450 monooxygenase (2E1 isoform) and a decrease in cytoplasmic HSP70 levels was observed.^142^ Both studies concluded that a rise in HSP60 levels is related to accumulation of misfolded proteins during periods of stress but ultimately this mechanism is surpassed by chronic damage and autoimmune processes may begin taking place.^142^

The onset of some forms of DCM are believed to be related to an autoimmune component that induces cardiac tissue remodeling through chronic inflammation. The increased expression of cell adhesion molecules observed in cells under stress make it possible for leukocytes to infiltrate into tissues following a chemokine gradient established by cells undergoing inflammation. A subtype of DCM, inflammatory DCM shows features similar to myocarditis and to the tissue remodeling and mechanical dysfunction of DCM. Immunohistochemistry of iDCM biopsies positive for CD3, CD45Ro, and CD68 are essential for diagnosis. To determine the mechanisms associated with the progression of iDCM, Bironaite et al.^150^ analyzed sera and biopsy samples from patients with this condition. Their results indicated a substantial rise in serum IL‐6, MMP9/TIMP1, HSP60 and caspases‐3, 8, and 9.^150^ Moreover, they found a correlation between serum IL‐6, the amount of infiltrated T lymphocytes (CD3^+^ enriched population) and secreted HSP60, suggesting that chronic inflammation induced by T cells and their secretion of IL‐6 compromises mitochondrial integrity and activity as observed by the induction of the apoptotic intrinsic pathway and the release of HSP60.^150^ It has also been determined the levels of serum antibodies to HSP60, HSP70, and heat shock cognate 71 kDa protein in patients with DCM, that were not otherwise observed in healthy control or coronary artery disease subjects, which provides an insight to support the autoimmune hypothesis before mentioned and the role of B cells in this process.^151^

HSP60 has been proven to be a cornerstone for mitochondrial metabolism regulation during induced insults in vitro but recent information has confirmed its paramount role in preventing DCM. Fan et al.^152^ recently developed an inducible cardiac‐specific HSP60 knockout mouse (HSP60CKO) model that exhibited the deleterious effects of HSP60 deletion in adult mouse cardiomyocytes. Authors generated inducible cardiac specific HSP60CKO mice with transgenic methods with the end result being tamoxifen inducible HSP60 downregulation in cardiomyocytes.^152^ From week 1 after tamoxifen injection the levels of HSP60 drastically dropped in cardiomyocytes and a full HSP60 level abolishment was achieved after 11 weeks of treatment.^152^ In contrast to controls all HSP60CKO mice died after 14 weeks of tamoxifen induction and histological analysis from heart recollection demonstrated ventricular dilation, wall thinning with extensive fibrosis and increase of cell apoptosis.^152^ Echocardiographic changes were recorded after week 9 with slightly decreased ejection fraction and by week 11 echocardiographic findings were consistent with histological results with enlarged left ventricle, reduced ejection fraction and wall thinning.^152^ Altered mitochondrial function was also demonstrated by spectrophotometric analysis and ROS levels measurement with results showing impairment of all four mitochondrial enzymatic complexes by week 11 and concomitant increases in ROS levels.^152^ With this new murine model Fan et al.^152^ were capable of recreating a more realistic pathophysiological system and similar lines of investigation may provide more reliable information of HSPs involvement during DCM development.

Finally, viral myocarditis, often linked to Coxsackievirus B3 (CVB3) infection, is known to be a predisposing factor for DCM, since the inflammatory response elicited during infection leads to a polyclonal expansion of immune cells even after the pathogen has been cleared, which may lead to autoimmunity. A chronic exposure to CVB3 has led to DCM through antibody‐mediated immunity, as IgM to antigens such as ɑ cardiac actin and HSP60 were found in sera of mice.^153^ These same antigens were found to be located close to the sarcolemma of myocytes as was observed by immunohistochemistry, while IgM antibodies had a similar distribution as indicated by immunoelectron microscopy.^153^ Altogether, this information remarks the importance of autocrine, paracrine and endocrine effects of HSP60 on cell homeostasis and disease progression.

### 8.8 Heart failure with preserved ejection fraction and diabetic cardiomyopathy

Hypertension is a common risk factor present in around 40%–60% of heart failure with preserved ejection fraction (HFpEF) patients which was historically referred to as diastolic HF in literature; however, recent pathophysiological understanding of the disease suggests other components for its development. Currently HFpEF attributes to nearly 50% of HF patients and its rising prevalence due to increasing comorbidities and lack of therapeutic options make it the perfect target for interesting new methods of investigation.

Left ventricle stress induced by hypertensive states has been studied as a potent inflammatory inducer. Mechanosensitive adhesion proteins such as adhesins and integrins have demonstrated to induce cellular inflammatory responses. IL‐18, TNF‐α, and IL‐6, and ANP can be induced in stretched myocytes and cyclic overload states have shown TLR4 upregulation.^154^ For this reason, Oyama et al.^92^ investigated the attenuation of LV hypertrophy progression during hypertensive state with the induction of HSPs in a murine model. Investigators compared mice with different diet induced hypertension regimens and added repetitive hyperthermia to certain groups.^92^ Results showed that fibrosis and cardiac hypertrophy were observed in the high salt diet group while these changes were not developed by the repetitive hyperthermia groups.^92^ Levels of HSP90, HSP70, and HSP60 were all elevated in repetitive hyperthermia mice and also measurement of inflammatory mediators such as TLR4, BNP, pentraxin related protein and thiobarbituric acid reactive substances were inhibited.^92^ Telomerase activity, telomeric DNA length and telomere reverse transcriptase were all preserved in repetitive hyperthermia groups.^92^ Conclusions from the study not only proved the anti‐inflammatory and antiremodeling properties of HSPs but also demonstrated that salt induced ventricular hypertrophy generates a marked inflammatory response in myocardium.^92^

In recent years emerging pathophysiological models are evidencing the systemic microvascular endothelial inflammation as a key factor for development of the condition. With these models all known causes of microvascular endothelial inflammation are recently recognized as independent risk factors; with obesity, diabetes mellitus, metabolic syndrome, lung diseases, smoking, and even iron deficiency being observed now as primary or secondary contributors. Inflammatory states seem to be initiated by multiple stressors with endothelial dysregulation being a paramount starting point. From here the increase in endothelial adhesion molecules and cytokines promotes monocyte migration. The consequences of macrophages inside the vessels and myocardium are an increase in ILs and other inflammatory mediators. Effects of multiple cytokines have been described and their effects on cardiomyocyte dysregulation are starting to emerge. IL‐1β and TNF‐α are notorious to cause dysregulation of calcium handling by the sarcoplasmic reticulum; leading to a negative inotropic effect. IL‐6 has been shown to reduce titin phosphorylation with increased cardiomyocyte stiffness. IL‐1β and TNF‐α also perform on cardiac fibroblasts upregulating angiotensin II type 1 receptors with fibrosis enhancement. Lastly, TNF‐α levels correlate with TGF‐β levels and its well‐known extracellular matrix effects. The endpoint of all these disturbances is increased stress to cardiomyocytes by inflammation and fibrosis, increased oxidative stress and alterations in cardiomyocyte signaling pathways. Ultimately slow LV relaxation and elevated diastolic left ventricle stiffness begin to appear.^154^, ^155^, ^156^ We group HFpEF and diabetic cardiomyopathy since the chronic inflammatory states of both diseases seem to fall in a spectrum of HF presentation.

In diabetic cardiomyopathy, it is well established that HSP60 molecules contribute as an important defense mechanism against hyperglycemic state‐induced apoptosis to cardiomyocytes. Although some of its benefits remain unknown, Chen et al.^157^ demonstrated a cardioprotective response from the interplay between HSP60 molecules and insulin‐like growth factor‐1 (IGF‐1). The study was carried out in streptozotocin (STZ)‐induced diabetic rats and it demonstrated that downregulation of HSP60 further decreased IGF‐1 receptor levels in diabetic myocardium and thus attenuated signaling in cardiomyocytes which in turn inhibited some of IGF‐1 cardioprotective mechanisms.^157^ After insulin treatment for 6 days augmented levels of both HSP60 and IGF‐1 was recorded.^157^ The direct cause of the reduced state in HSP60 levels in diabetic myocardium is not yet fully identified. Shan et al.^158^ demonstrated that continuous cardiomyocyte stimulation with high glucose induced specific upregulation of microRNAs miR‐1/miR‐206 with posttranscriptional HSP60 regulation. Further experiments need to address the complete mechanisms of HSP60 downregulation to provide new therapeutic assessments of the HSP60 protective properties, however, the reduction of intracellular HSP60 continues to have a deleterious effect on cellular function and denotes that stressors such as a diet high in salt and glucose, coupled with a western diet rich in cholesterol, deregulate these highly specialized signaling pathways affecting homeostasis.

### 8.9 Pulmonary hypertension

Pulmonary arterial hypertension (PAH) as a pathological entity involves a varied list of etiologies and its complete pathophysiological mechanisms are not fully understood. From chronic pulmonary insults to idiopathic or even venous system pathologies, PAH involves a continuous elevation of pulmonary vascular resistance. The end result of such insult is right ventricular remodeling and ultimately right HF.

The relationship between PAH pathogenesis and HSPs has been demonstrated by recording elevated levels of some HSPs in PAH patients. Several authors have correlated the appearance of some HSPs as a safeguarding mechanism against stress which promotes resistance to apoptosis and proliferation of VSMCs.^159^ A study demonstrated that broiler chicken with triiodothyronine‐induced pulmonary hypertension initially compensated disease development with the upregulation of mRNAs of several HSPs including HSP60, HSP90, and HSP70.^160^ RT‐PCR on tissue from right heart ventricles was performed at days 12 and 42 with the former showing upregulation of HSPs and the latter with decreased amounts of such proteins in relation to control groups.^160^ Authors came to the conclusion that HSPs may be upregulated in an attempt to postpone the pathological processes of the disease and its considerable reduction may implicate a declining compensatory response of failing dilated hearts.^160^

Furthermore, the inhibition and downregulation of HSP90 and HSP70 respectively has proven to be effective in some experimental models of PAH.^161^, ^162^ It seems that the blockage of HSP90's activity by 17‐allylamino‐17‐demethoxygeldamycin, 17‐AAG, translates to an improvement in pulmonary arterial remodeling manifested as decreased pulmonary artery pressures and lack of right ventricle hypertrophy.^161^ Findings evidenced reduced wall thickness overall, which in turn could be a direct consequence of the lack of the arrest of cell cycle progression by inhibiting HSP90 and suppression of PDGF‐induced proliferation of VSMCs.^161^ In another study, Boucherat et al.^159^ demonstrated that gamitrinib, a mitochondrial targeted HSP90 inhibitor, reduced survival mechanisms in pulmonary artery VSMCs with subsequent improvement of PAH. The presence or absence of these HSPs is important as they interact with HSF‐1, a transcription factor of HSPs, which influence the regulation of coexpression of HSP60.^163^ Treatment with proanthocyanidin (GSP), a natural grape seed extract, induced beneficial effects in PAH.^162^ GSP downregulates expression of HSP70 which reduces expression levels of pho‐IκBα.^162^ As an activator of NF‐κB, low levels of pho‐IκBα promotes less NF‐κB phosphorylation, hindering proliferation, and growth of VSMCs.^162^ With current therapeutic strategies being focused mostly on vasodilation and anti‐inflammatory actions, PAH continues to be a progressive and lethal disease. Promising results from regulating HSPs and newer lines of investigation will eventually replace the common therapeutic options against PAH, with anti‐remodeling strategies promising to be a mainstay.

**Figure 2:**
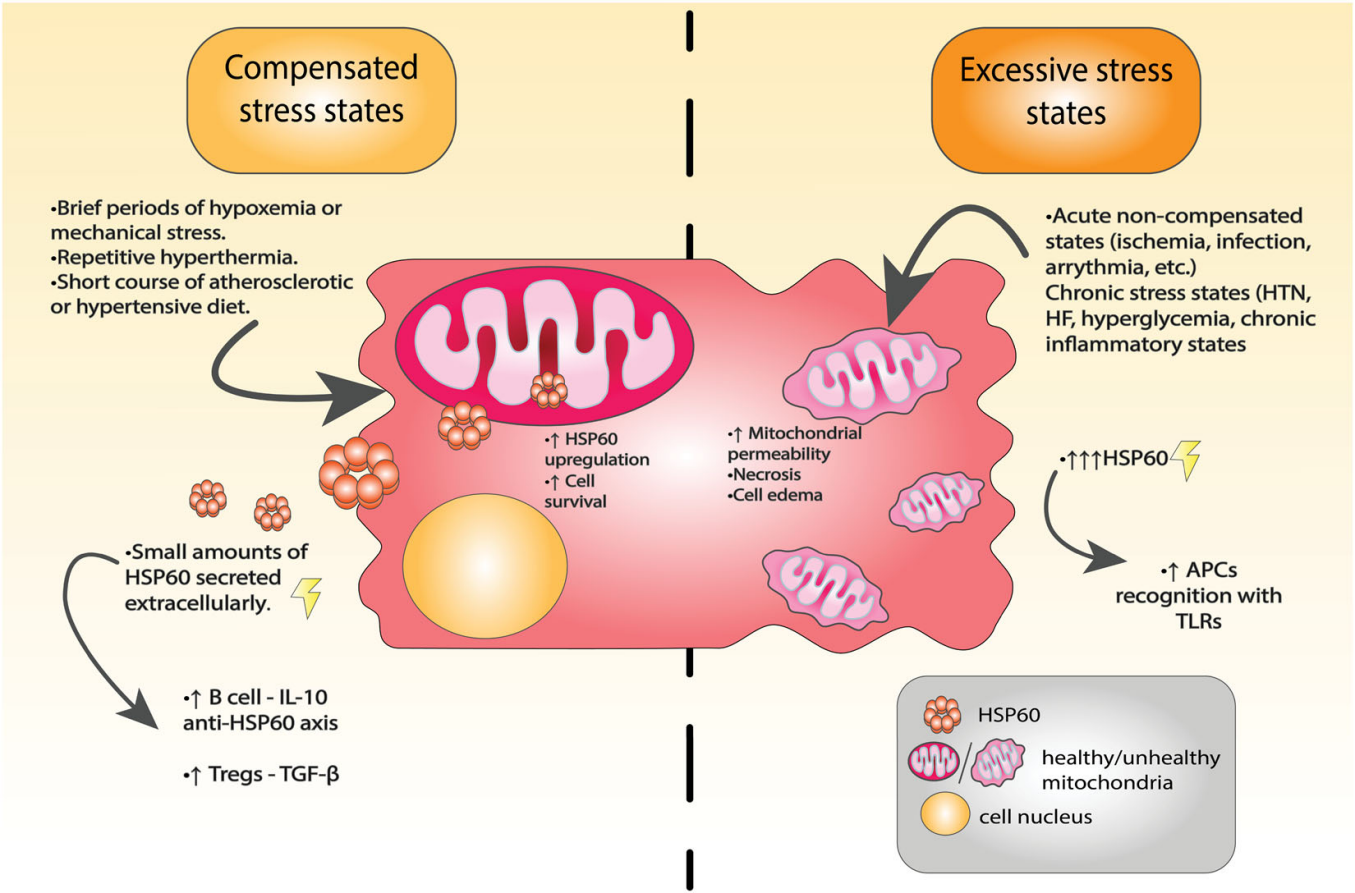
Compensated stress states vs excessive stress states. HSP60 works in a bimodal fashion depending on the insults involved. Left half of the image: compensated stress states render a favorable mitochondrial adaptation and upregulation of HSP60 levels. With these upregulated chaperones the cardiomyocyte survival is increased. Note that small amounts of HSP60 molecules are also excreted with the help of exosomes, initiating the anti‐HSP60 and Tregs response = anti‐inflammatory effect. Right half of the image: Acute excessive stress alters several components of cell survival with the net effect being mitochondrial and cell swelling and increased permeability. This increases HSP60 levels in the extracellular space dramatically (with other necrosis markers being exposed also). HSP60 acts as a potent APC activator extracellularly increasing inflammation and remodeling of tissue. APC, antigen presenting cell [Color figure can be viewed at wileyonlinelibrary.com]

As a brief summary of interpretation and for better understanding we developed the following model of interaction between HSP60 and the above‐mentioned insults (Figure 2). Establishment of the complete mechanisms of these effects is yet to be elucidated.

## 9 THERAPEUTIC STRATEGIES TARGETING THE HSP60 SIGNALING PATHWAY

**Figure 3:**
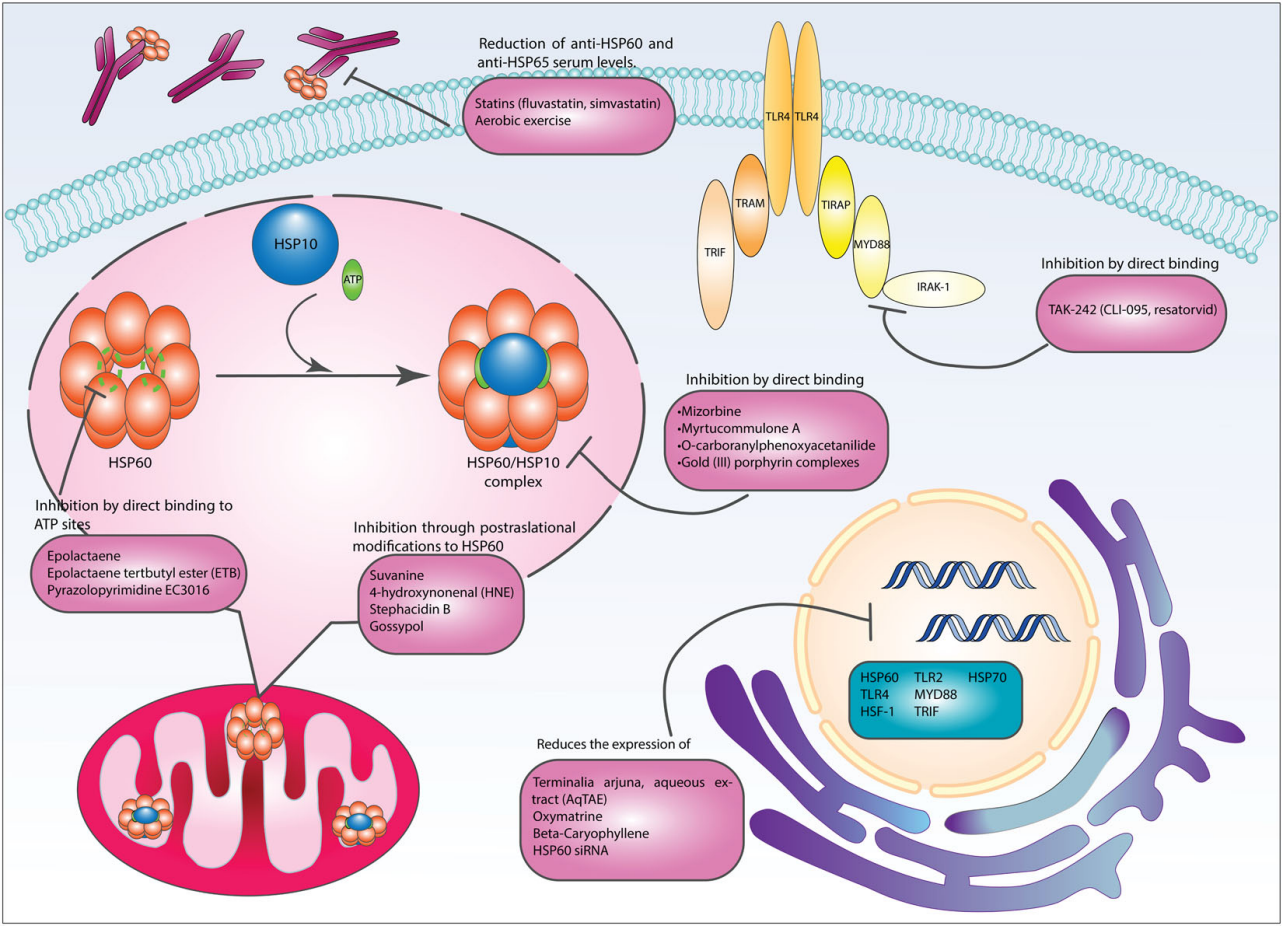
Therapeutic strategies targeting the HSP60 signaling pathway. Small molecular inhibitors of natural and synthetic origin modulate HSP60's structure, expression, folding activity, and titers of anti‐HSP60 immunoglobulins. TLR4 is also a target for drugs inhibiting the binding of downstream adaptor proteins. Therapeutic interventions are grouped according to their mechanism of action [Color figure can be viewed at wileyonlinelibrary.com]

Since the emergence of interesting findings regarding HSP60 as a mitochondrial chaperone, studies have consistently shown that its localization can also extend to outside the mitochondria performing both non‐chaperoning and chaperoning roles. As aforementioned, accumulating data has evidenced that HSP60 is present in different stages of CVD, which exhibit impaired concentration levels of HSP60. Gathering evidence of HSP60 as a signaling molecule and an inflammation elicitor and reviewing all the previous findings in different CVDs, a clear pathway to elaborate potential therapies is demarcated by targeting this chaperonin and other proteins related to the various downstream effects it triggers (Figure 3).

In this regard, studies have shed light to some small molecule modulators for this protein. Some are natural molecules and others are synthetic entities with uncommon pharmacophores or structural motifs with the capacity to modulate its function (Table 2). In the following section we highlight the recent studies done in this particular area of interest with reported modulating agents and inhibitors.

**Table 2:** Small molecular inhibitors targeting HSP60 and TLR4

Strategy	Molecular nature	Mechanism of action	Tested on	Reference
*Anti‐HSP60*				
Mizorbine	Imidazole nucleoside antibiotic from	Blocking of ATPase activity at the HSP60‐ HSP10 complex through direct binding	T cells	^164^
	*Eupenicillium brefeldianum*			
Epolactaene	From *Penicillium* spp.	Inhibition of HSP60 and HSP10 through binding to Cys442 residue at the ATP‐binding site	SH‐SY5Y cells	^165^, ^173^, ^210^, ^211^
Epolactaene tertbutyl ester	Structural modification from epolactaene	Allosteric modulation of HSP60‐HSP10 through covalent binding to Cys442	SH‐SY5Y cells	^168^, ^172^, ^173^, ^174^, ^175^
		Inhibition of ATPase activity after binding to Cys138 in GroEL		
*Terminalia arjuna*, aqueous extract	Aqueous extract of *T. arjuna*	Reduction of expression levels of HSP60 and HSP70	Rabbits	^180^
Oxymatrine	Alkaloid derived from *Sophora flavescens*	Reduction of protein expression levels of HSP60, HSF‐1, and TLR4	BV2 microglial cells	^181^, ^182^, ^183^, ^184^
Myrtucommulone A	Nonprenylated acylphlorogluricinol	Blocking of protein folding activity at the HSP60‐HSP10 complex through direct binding	Isolated mitochondria from human leukemia cells	^165^, ^166^, ^167^
β‐Caryophyllene	Natural product present in cinnamon, cloves, basil, and black pepper	Reduction of protein expression levels of TRIF, MYD88, HSP60, TLR4, and TLR2	Isoproterenol‐induced myocardial infarction model	^185^
Suvanine	Natural sesquiterpene of marine origin	Sulfation of residues of cysteine in HSP60	Proteomic screening interactions	^176^
4‐Hydroxynonenal	α, β‐unsaturated hydroxyalkanoate product from lipid peroxidation in cells	Binding to HSP60 after nucleophilic attack of cysteine thiol group on the electrophilic α, β‐unsaturated aldehyde moiety from HNE	Proteomic analysis	^165^
Stephacidin B	Natural product isolated from *Aspergillus ochraceus* WC76466	Alkylation of the thiol groups in HSP60 through the 3‐alkylidene‐3H‐ indole 1‐oxide electrophilic moiety	Cancer cells	^164^, ^177^, ^178^
Avrainvillamide	Natural product isolated from *Aspergillus* spp. CNC358	Alkylation of the thiol groups in HSP60 through the 3‐alkylidene‐3H‐ indole 1‐oxide electrophilic moiety	Cancer cells	^164^, ^177^, ^178^
O‐carboranylphenoxyacetanilide	Synthetic molecule	Blocking of ATPase activity at the HSP60‐HSP10 complex through direct binding	HeLa cells	^168^, ^169^
Gold (III) porphyrin complexes	Synthetic compound	Blocking of protein folding activity at the HSP60‐HSP10 complex through direct binding	Thermal shift assays, chemoproteomic and saturation‐transfer difference‐nuclear magnetic resonance (STD‐NMR) in cells	^170^
Statins (fluvastatin, simvastatin)	Lipid‐lowering drugs	Lowering anti‐HSP60 and anti‐HSP65 serum levels	Patients during the rehabilitation period after percutaneous intervention due to unstable angina	^188^
Aerobic exercise	Nonpharmacological intervention	Lowering anti‐HSP60 and anti‐HSP65 serum levels	Patients during the rehabilitation period after percutaneous intervention due to unstable angina	^188^
Gossypol	Polyphenolic drug	Inhibits the thiol/disulfide redox reactions from HSP60's cysteine residues through direct interaction	Cancer cells	^179^
Pyrazolopyrimidine EC3016	Aromatic heterocycle	Blocking of protein folding activity at the HSP60‐HSP10 complex through blocking of ATP binding sites and hydrolysis	Purified *GroEL*	^171^
HSP60 siRNA	eGFP conjugated siRNA	Reduction in HSP60 and associated protein levels	N9 microglial cells	^68^
*Anti‐TLR therapies*				
TAK‐242, CLI‐095, resatorvid	TLR4‐specific inhibitor	Blocks binding of IRAK‐1 to TLR4.	RAW264.7 cells, rats	^68^, ^189^, ^190^, ^191^
		Inhibition of IRAK‐1		

*Note:* Mechanism of action and source different molecules tested.

Abbreviations: eGFP, enhanced green fluorescent protein; HSF‐1, heat shock factor‐1; HSP, heat shock protein; IRAK‐1, interleukin‐1 receptor‐associated kinase 1; MYD88, myeloid differentiation primary response 88; siRNA, small interfering RNA; TLR, toll‐like receptor; TRIF, TIR‐domain‐containing adapter‐inducing interferon‐b.

### 9.1 Anti‐HSP60 therapies

As described throughout this review, the HSP60‐related cardiovascular burden encompasses several pathophysiological mechanisms and targets while it also plays a crucial part in different diseases. Developing modulators targeting HSP60 are potentially useful as therapeutics as blockage of HSP60 halts posterior inflammatory cascades to flare up in the myocardium.^123^ Although many natural and synthetic molecules have been formulated to target other chaperones, only a handful have been developed aimed toward HSP60, making it a novel and innovative target. The known HSP60 inhibitors are conventionally classified according to their mechanisms of action into two main categories: type I and type II inhibitors. According to Meng et al. and Palumbo et al., type I inhibitors participate in ATP binding and hydrolysis, thus affecting HSP60's reactions crucial for protein folding.^164^, ^165^ Some reported members of this group include naturally occurring molecules such as: (1) mizoribine, an imidazole nucleoside from *Eupenicillium brefeldianum* ^164^; (2) myrtucommulone A, a non‐prenylated acylphloroglucinol found in myrtles, a class of evergreen shrub found along the Mediterranean.^165^, ^166^, ^167^ The synthetic arm of type I inhibitors consists of the following known molecules: (1) O‐carboranylphenoxyacetanilide, which shows strong selectivity for HSP60 over other chaperonins^168^, ^169^; (2) Gold (III) porphyrin complexes, that allows for binding to its target by means of both electrophilic and hydrophobic interactions^170^; (3) pyrazolopyrimidine EC3016, an aromatic heterocycle that has so far only been described in relation to its HSP60 inhibitory activities.^171^ On the other hand, type II inhibitors target cysteine residues in HSP60 for covalent binding or oxidative modifications likely by reacting with an electrophilic moiety on drugs from this group. Most of the molecules identified from this group are of natural origin, and these include: (1) Epolactaene and epolactaene tert‐butyl ester, isolated from *Penicillium* spp. Both of them exert their effects by binding to a Cys442 residue on HSP60, but only epolactaene tert‐butyl ester interferes with its ATPase through what appears to be an allosteric modulation^168^, ^172^, ^173^, ^174^, ^175^; (2) Suvanine, a sesquiterpene isolated from a *Coscinoderma* sp. sponge from the micronesian islands that modifies the chaperonin's structure by targeting its cysteine residues for sulfation^176^; (3) Stephacidin B and avrainvillamide, both isolated from different strains of *Aspergillus ochraceus*, WC76466 and CNC358 respectively. These molecules also induce posttranslational modifications by alkylating thiol groups on the chaperonin, although more research is needed to support their overall effect on the protein's activity^164^, ^177^, ^178^; (4) Gossypol, a phenolic aldehyde present in the cotton plan (Gossypium) also targets thiol groups and affects HSP60's redox potential^179^; and lastly (5) 4‐hydroxynonenal, an advanced lipid peroxidation end product, is suggested to cause structural modifications from nucleophilic attacks of HSP60's cysteine thiol groups on the electrophilic aldehyde groups in 4‐hydroxynonenal.^165^

Molecules that modulate HSP60 at protein expression levels have also been reported and mainly include compounds from plants such as the aqueous extract of the tree *Terminalia arjuna* ^180^; oxymatrine from the chinese herb Sophora flavescens,^181^, ^182^, ^183^, ^184^ and; β‐caryophyllene, found in essential oils of various plants such as cinnamon, basil, cloves, and black pepper.^185^ Molecular biology constructs such as siRNA have also been developed and have proven to be successful in decreasing HSP60's activity.^68^ These molecules work by downregulating the expression of not only HSP60 but also other related proteins such as TLR4, HSF‐1, MYD88, TIR‐domain‐containing adapter inducing interferon b and proinflammatory cytokines, to mention a few examples. Hu et al.^186^ analyzed the beneficial effects of carvedilol, a nonselective β‐blocker, associated with microRNA‐1 (miR‐1) regulation in a rat model of AMI by coronary artery occlusion. MiR‐1 is deemed an important regulator in cardiac development and disease, more specifically, in the regulation of cardiomyocyte apoptosis in the latter.^186^ They observed how miR‐1 overexpression was associated with decreased cell viability and how the effect of carvedilol is to precisely counteract this result via downregulation of miR‐1 and thus exercising an antiapoptotic reaction.^186^ In an effort to further analyze this mechanism, expression of HSP60 was measured, a target of miR‐1.^186^ Carvedilol was able to increase mRNA expression levels of HSP60 while simultaneously downregulating miR‐1 expression.^186^ The resulting upregulation of HSP60 was linked to an upregulation of Bcl‐2 and downregulation of Bax.^186^ A key step through which HSP60 protects cardiomyocytes from apoptosis, is the formation of complexes with Bax thus inhibiting Bax oligomerization and insertion in the mitochondrial membrane and evading apoptosis.^41^ An additional benefit of targeting HSP60 is its potential use to curb tumor cell growth and survival. In some kinds of tumors where overexpression of HSP60 helps cancer cells flourish, this type of chaperonotherapy aid in activating apoptosis in cancer cells without obstructing normal HSP60 functions in other cells. Pace et al.^187^ have further revised anti‐HSP60 treatment as cancer therapy and recapitulated the molecular mechanisms behind.

Lastly, the reduction of serum levels of anti‐HSP60 and anti‐HSP65 Igs is another attractive therapeutic ally, since these also cause systemic damage by activating the complement cascade and specific receptors immune cells. In this regard, some lipid lowering drugs from the family of statins, such as simvastatin and fluvastatin, have been reported to lower HSP60‐specific Ig seric levels although this observation is likely independent from their hypolipidemic and anti‐inflammatory effects. Aerobic exercise is interesting in that it is a nonpharmacological strategy that has also shown to reduce seric anti‐HSP60 Ig levels, suggesting another mechanism by which physical activity may be beneficial for various diseases.^188^

### 9.2 Anti‐TLRs therapies

An alternative to the use of anti‐HSP60 for therapeutic purposes is inhibiting TLRs, since these play a predominant role in the pathway for HSP60‐induced inflammatory response. Specifically, TLR4 genetic deletion has proven to attenuate inflammation in the myocardium during ischemia/reperfusion.^123^ Two studies described that TAK‐242 (resatorvid) also known as CLI‐095, a specific inhibitor of TLR4 signaling acts by blocking the interaction between activated TLR4 and its adaptor intracellular molecule IRAK‐1, thus impairing NF‐kB activation.^189^, ^190^ TAK‐242 was also tested as a therapeutic treatment by Abdul et al.,^191^ where TLR4 inhibition showed a beneficial role in preventing amplified neurovascular injury on diabetic rats after acute ischemic stroke. Another study demonstrated that the stimulation of endogenous IRAK‐1 in RAW264.7 cells was inhibited by TAK‐242.^190^ To inhibit the proinflammatory response of HSP60 and the TLR4 downstream signaling, the usage of CLI‐095 showed significantly decreased levels of iNOS, COX‐2, proinflammatory cytokines, activation of p38, and levels of IL‐1β‐induced inflammatory molecules.^68^

### 9.3 Tolerization strategies

The immune system clears pathogens by responding to antigen stimulation under normal circumstances, this is referred to as the positive immune response. In contrast, the immune system may develop “unresponsiveness” specific to certain antigens after antigen stimulation, which is referred to as immunological tolerance (immune tolerance) according to Sun et al.,^192^ and as a matter of fact, tolerization is defined as the induction of immunological tolerance. It is proposed that HSP60 administration can act as an immunologic modulator for prevention and treatment of different diseases such as the ones mentioned before in this review.

Vaccination strategies that have proven to be successful in inducing a tolerogenic state usually follow oral or nasal administration routes (Table 3). In these approaches, small concentrations of an antigen to which tolerance is sought are employed for immunization. Most of the current evidence of the potential of these strategies in treating CVDs have been tested on atherosclerosis due to its strong immune pathophysiological component. Several studies conducted using either oral or nasal immunization protocols have demonstrated to reduce the size of atherosclerotic plaques, to reduce the number of new plaques and to improve the overall outcome of treated animals in atherosclerosis disease models induced by high fat and cholesterol diets in genetically susceptible ApoE^−/−^ or LDL^r−/−^ mice.^193^, ^194^, ^195^ These biological effects observed after immunization have been associated to systemic and localized (at the site of lesions) cellular and molecular changes characterized by a shift from Th1/Th17 to Th2 mediated actions with the corresponding modulation of their associated cytokines, decreased macrophage recruitment and activity in the atherosclerotic plaques, and induction of immune suppressing cells such as Tregs and myeloid derived suppressor cells (MDSC) with CD4^+^CD25^+^Foxp3^+^ and CD11b^+^ as their commonly associated phenotypic markers, respectively.^194^, ^196^

**Table 3:** Immunization strategies inducing tolerization to HSP60

Strategy	Components	Molecular mechanism	Biological effect	Tested on	Reference
*Tolerization*					
Vaccine development	Cholera toxin (CTB), IL‐4 and TTFrC (helper epitopes) prompting epitopes (four HSP60 and two calreticulin peptides)	Shift from Th1 to Th2 response with secretion of associated cytokines	Not applicable	In silico	^199^
Oral or subcutaneous immunization	HSP60 administration	Oral HSP60 increased the levels of CD11b^+^, Gr‐1^+^ MDSCs in peripheral blood with increased mRNA levels of Arg1, iNOS	Oral HSP60 suppressed atherosclerotic lesions	ApoE^−/−^ mice fed on a Western‐type diet	^193^
			Subcutaneous HSP60 aggravates atherosclerosis		
		Subcutaneous HSP60 increases the expression of ROR‐γt cells			
Oral immunization	Oral HSP60 administration	Induction of CD4^+^CD25^+^Foxp3^+^ Tregs	Reduction of atherosclerotic lesions	ApoE^−/−^ mice	^194^
Oral immunization	Vaccine using oxLDL and HSP60 antigens	Increased concentration of Foxp3^+^ Tregs in some organs	Decrease in atherosclerotic plaque formation	LDLr^−/−^ mice	^197^
Nasal or oral immunization	Mycobacterial HSP65 administration	Decreased presence of macrophages, CD4^+^ T cells and IFN‐γ	Decrease in atherosclerotic plaque formation	LDLr^−/−^ mice	^195^
		Increased production of TGF‐β and IL‐10 by lymph nodes			
Nasal immunization	HSP60 administration	Increase in CD4^+^CD25^+^, CD4^+^LAP, TGF‐β, and Foxp3^+^ Tregs	Suppression of atherosclerosis	Mice	^196^
Nasal immunization	HSP60/65 administration	Increased number of CD4^+^CD25^+^ GARP^+^ Tregs in cervical lymph nodes and spleen. Increased TGF‐β, Foxp3, IL‐10, and Tr1.	Reduction of atherosclerotic lesions	ApoE^−/−^ mice	^194^
		Decreased levels of Th1 and Th17 cells			
Oral immunization	Vaccine using HSP60 peptide 153‐163 and ApoB‐100 peptide 661‐680	Increased levels of TGF‐β and induction of CD4^+^CTLA^+^CD25^+^Foxp3^+^ Tregs	Reduction of atherosclerotic lesions	ApoB^tm2Sgy^LDLr^tm1Her^/J mice	^198^
Oral immunization	Vaccine using peptides from human HSP60, ApoB‐100 and the outer membrane protein of *Chlamydia pneumoniae*	Activation of CD11c^+^ and CD103^+^CD11b^+^ cells and Tregs in gut lymphoid organs	Reduction of atherosclerotic lesions	ApoB^tm2Sgy^Ldlr^tm1Her^/J mice	^212^

*Note:* Experimental evidence on the cellular and molecular changes observed after immunization with HSP60, selected peptides or in association with other antigenic sequences through different administration routes. Biological effect on in vivo atherosclerosis models is also indicate.

Abbreviations: iNOS, inducible nitric oxide synthase; TGF‐β, transforming growth factor β.

Immunization using HSP60 as an antigen can be carried by administering the whole protein or selected disease‐inducing peptides to which tolerization is desired; this last approach accounts for more tailored vaccines inducing tolerance to sequences of interest while sparing immunogenicity for the rest. Oral immunization using peptides from HSP60 together with some from other atherosclerosis‐related antigens such as oxLDL and ApoB‐100 have also been tested demonstrating effective induction of tolerance featuring the cellular and molecular hallmarks aforementioned; moreover multi‐antigenic vaccines using more than two antigenic sources are yet another take on tolerization that is being explored in the search for higher specificity responses.^197^, ^198^ In silico modeling is also helpful in predicting the immunogenic and tolerogenic potential of selected antigens for vaccine development and optimization,^199^ which makes it a highly valuable tool for future therapeutics of this nature.

### 9.4 Other strategies

Innate immunity originally evolved as an immediate protective response to foreign antigens, as induction of antibodies takes 4–5 days. However, this important part of the immune system has a dark side, as it also responds to the release of common endogenous proteins into the extracellular space. As previously mentioned, a rising number of evidence points toward the damaging role played by DAMPs released into the extracellular space after MI, where they may activate PRRs and promote inflammation in the surrounding affected tissues, which may serve as a bona fide mechanism for macrophage recruitment and clearance of necrotic and apoptotic cell debris, as well as activation of myofibroblasts to produce granulation tissue that allows for local repair. Nevertheless, failure to contain the inflammatory response leads to continuous remodeling of the affected tissues, which explains the progressive changes in the left ventricle following damage by MI. Thus, inflammation is a hidden contributor to the development of HF. In this regard, some studies have discovered other potential therapeutic strategies that influence the inflammation agenda. In a study that shed light on the mechanisms behind cardiac dysfunction post‐AMI, the role of the protein phosphatase Mg^2+^/Mn^2+^–dependent 1L (PPM1L) in post‐AMI inflammation and LV remodeling was studied in vivo.^200^ PPM1L transgenic mice underwent sham or ligation of the left anterior artery operation for AMI model.^200^ HSP60 and HMGB1 release was identified in myocardial tissue after day 1 or 3 posterior the ligation, and as DAMPs for TLR4 trigger TLR‐mediated inflammation, it is a potential cause for the progression of cardiac dysfunction.^200^ Data evidenced that PPM1L causes a significant downregulation of TLR‐mediated inflammation and cytokine production by macrophages. Also, PPM1L binds directly with IKKβ, an important kinase of NF‐κB, hindering activation of the NF‐κB signaling pathway. Thus, their data suggests that in the presence of DAMPs in an AMI model, PPM1L acts as a negative regulator of postinflammatory processes that exacerbate myocardial damage acting as a protective protein in this disease.^200^

Another group studied Kelch repeat and BTB domain‐containing protein 7 (KBTBD7) and miR‐21 as novel markers associated to inflammation and development of innate immune responses to DAMPs, using HSP60 as a prototype, in an in vivo mouse model of CAL‐induced AMI.^201^ Their model poses miR‐21 behaving as a negative regulator of HSP60‐induced inflammation, where a broader proinflammatory response and worse overall outcome after AMI was observed for miR‐21_KO_ mice, which was associated with an increased release of cytokines such as TNF‐α, IL‐6, and IL‐1β by cardiac CD11b^+^ macrophages.^201^ Moreover, it was found that said cellular events were related to increased phosphorylation of different proteins of the MAPK and NF‐κB pathways, including p38, IKKα/β and p65, which were markedly present also in miR‐21_KO_ mice.^201^ Using bioinformatic tools for identifying microRNA biological interactions, KBTBD7 was suggested to have a target site for miR‐21, and luciferase reporter assays demonstrated the inhibitory role this noncoding RNA has when bound to it.^201^ Further investigation of the functional relation of KBTBD7 on HSP60‐induced inflammation revealed that this protein may enhance the activity of MKK3/6, an upstream kinase for MAPK and NF‐κB phosphorylation, which suggests an intricate interplay between KBTBD7 and its regulation by miR‐21.^201^ Thus, the result was preventing cardiac remodeling and scar formation, preserving cardiac function post‐AMI. These previous studies attained such results by directly or indirectly blocking excessive inflammation at different regulatory points, such as activation of NF‐κB and MAPK pathways, TLR‐mediated inflammation and cytokine production, controlling the overall outcome of myocardial infarction.

## 10 CONCLUSIONS

According to the World Health Organization, CVDs occupy first place in morbidity and mortality worldwide. It is estimated that by the year 2020, deaths from CVD will increase by 15%–20% and about 23.6 million people will expire from these disorders by 2030, mainly from stroke and heart disease. The beginning and development of these pathologies are intimately related to proinflammatory mechanisms occurring at different structural levels leading to the development and progression of cardiovascular damage accompanied by acute or sustained production of proinflammatory cytokines. Indirectly and acutely, these proinflammatory cytokines can depress the contractility of cardiomyocytes, by affecting the response to β‐adrenergic stimuli thereof, by modulating NO activation mechanisms. As we discussed in this study, some of the molecules that have aroused great interest for its participation in autocrine or paracrine effects and in the modulation of the immune system and its possible capacity to act as a marker of cardiovascular cellular damage are the HSPs. In particular, HSP60 plays an important role by its function as a modulator of the innate and adaptive immune response. It has been shown that in patients with several CVDs, HSP60 presents an abnormal cellular distribution, localized in the cell membrane, which has been correlated with an increase in cell death by apoptosis. On the other hand, it has been shown that the presence of extracellular HSP60 activates the immune response facilitating a systemic proinflammatory state, with a rise in TNF‐α production and other proinflammatory mediators that perpetuate the progression of HF.

This dual role of HSP60 as an immunomodulator and biomarker of damage allow us to explore the potential therapeutic options in a short and medium term. Until now, it has been evident that the modulation of the immune system by means of antigenic molecules could be established as a powerful curative strategy. Novel treatments may target the inhibition or stimulation of inflammatory mediators to induce a specific response on various inflammatory biomarkers and the inactivation of modulators of cell damage and death. This specialized area targeting specifically HSP60 in CVD seems to be still fresh and yet to be looked into deeply. Therefore we believe further interest will be garnered in this field as various studies mentioned earlier describe promising results exploiting the capacity to trigger or mitigate inflammation, as well as the regulation of expression levels of HSP60, while taking into account the functions and effects it elicits, all of which could be used at different stages of disease progression in CVDs where studies reveal different patterns of expression levels of HSP60 as well as the participation of various inflammatory components and other HSPs members, all of which are well established direct and indirect targets of HSP60. As promising as it looks, much more is yet to be understood and a long path lies ahead with unexplored terrain to be covered.

## CONFLICT OF INTERESTS

The authors declare that there are no conflict of interests.

## AUTHOR CONTRIBUTIONS

IKS and IAMR searched literature; IKS, IAMR, RAVL, and CEGB conceptualized and drafted the manuscript; IKS, GTA, AAK, and CEGB revised the manuscript; IKS, AAK, and CEGB discussed the manuscript.
